# Hydrazinoacetic acid is a biosynthetic precursor of the bacterially produced nitramine, *N*-nitroglycine

**DOI:** 10.1128/aem.00631-26

**Published:** 2026-05-20

**Authors:** Gabriel Padilla, Benjamin M. Rathman, Shivaiah Vaddypally, Brenda Martinez Rodriguez, Michael J. Zdilla, David E. Graham, Jonathan D. Caranto

**Affiliations:** 1 Department of Chemistry, University of Central Florida 6243, Orlando, Florida, USA; 2 Department of Chemistry, Temple University 6558, Philadelphia, Pennsylvania, USA; 3 Biosciences Division, Oak Ridge National Laboratory 6146, Oak Ridge, Tennessee, USA; Kyoto University, Kyoto, Japan

**Keywords:** biotechnology, biosynthesis, energetics, nitramine, natural product, secondary metabolism, energetic material

## Abstract

Nitramines [R(R′)N–NO_2_; R,R′=H or alkyl] are valuable synthetic products, but knowledge of the biosynthetic processes that generate these compounds is limited. This work sought to elucidate the biosynthesis of a nitramine natural product, *N*-nitroglycine (NNG) by *Streptomyces noursei*. Stable isotope studies showed that *S. noursei* cells supplemented with l-(ε-^15^N)lysine, (^15^N)glycine, or (^13^C)hydrazinoacetic acid (HAA) incorporated 67%, 88%, and 67% of the isotope label into NNG, respectively, indicating that these compounds are biosynthetic precursors of NNG. Liquid chromatography coupled tandem mass spectrometry (LC-MS/MS) of ^15^N-Lys-labeled NNG confirmed that the nitro nitrogen of NNG originates from Lys. Bioinformatics analysis of the *S. noursei* genome showed evidence for a biosynthetic gene cluster (BGC) that contained machinery for HAA biosynthesis (*nngKLM*), consistent with the results of the isotope labeling. *In vitro* reconstitution of the gene products produced HAA. The borders of this BGC were defined by cross-referencing the predicted BGC with previously published differential proteomics data. Furthermore, we show that azaserine is produced alongside NNG in *S. noursei* cultures, linking the two biosynthetic pathways via a proposed nitrosamine biosynthetic intermediate. Finally, the oxygen balance for NNG is −20.2% for the formation of carbon dioxide (CO_2_), which is comparable to that of hexahydro-1,3,5-trinitro-1,3,5-triazene (common name: RDX; −21.6%). Crystal structure data of NNG indicate that the unit crystalizes as a pure material, not a hydrate, suggesting a favorable energetic crystallization phase. The combined results suggest a route that, with further development, could lead to sustainable production of energetic nitramines via synthetic biology or biocatalytic approaches.

**IMPORTANCE:**

Chemical manufacture of nitramine energetics produces hazardous waste streams. Mitigation of these waste streams could be accomplished by replacing current production processes with enzymatic or synthetic biology approaches, enabling sustainable production of these compounds. This study identifies a *Streptomyces noursei* biosynthetic gene cluster that produces a nitramine natural product, *N*-nitroglycine, via the formation of a hydrazine precursor. The combined data, along with previously reported proteomics data, define the boundaries and genetic inventory of the biosynthetic gene cluster. Furthermore, structural and thermal degradation analyses of the natural product suggest favorable properties for the use of *N*-nitroglycine, itself, as an energetic material. Further development of this pathway could enable sustainable approaches to produce energetic nitramines either by oxidation of non-native polyhydrazines to the corresponding polynitramines or by improving production and isolation of *N*-nitroglycine from *S. noursei* cultures.

## INTRODUCTION

**Fig 1:**
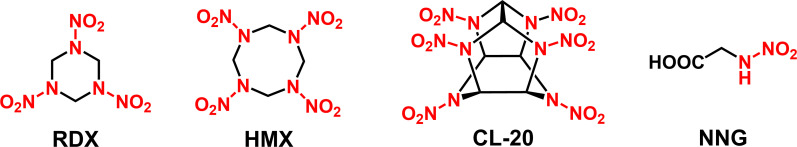
Nitramine compounds, including the energetic compounds RDX, HMX, and CL-20, and *N*-nitroglycine (NNG), a natural product nitramine produced by *S. noursei*, are shown.

Nitramine functionalities [R(R′)N–NO_2_; R,R′=H or alkyl] are critical explosophores for several high-energy compounds, including the energetic materials hexahydro-1,3,5-trinitro-1,3,5-triazene (RDX), octahydro-1,3,5,7-tetranitro-1,3,5,7-tetrazocine (HMX), and hexanitrohexaazaisowurtzitane (CL-20) (Fig. 1). These compounds are explosives and propellants used as components of a wide range of military munitions, ordnance, and propellants (1). Manufacture of these compounds requires corrosive fuming nitric acid, acetic acid, and organic solvents, thereby generating several hazardous waste streams (2). In addition, these reactions are highly exothermic and require extensive purification processes due to their poor product specificity and regioselectivity (3).

Biological manufacturing of nitramines using enzymes could eliminate some of the waste generated by these manufacturing processes (4). Enzymatic reactions are performed under mild conditions, including ambient temperatures and pressures, and within aqueous solvents. Furthermore, enzymes are usually regioselective and form specific products. Therefore, developing an enzymatic process for nitramine production would allow for more sustainable manufacturing of energetic compounds and precursors via biocatalytic or synthetic biology approaches. However, enzymes that can install nitramine functionalities are unknown.

One potential route toward identifying such enzymes is to elucidate the biosynthesis of nitramine natural products. *N*-Nitroglycine (NNG), a natural product, is the only known nitramine produced by bacteria (5). This NP was discovered in the cultures of *S. noursei,* and it was first reported in 1968 (6). While there has been progress characterizing NNG biosynthesis (7, 8), the pathway has not been completely elucidated.

Prior studies provided some insight into this pathway (8). Mass spectrometry was used to monitor NNG production by *S. noursei* from its [M–H]^–^ peak at *m*/*z* 119. When *S. noursei* cultures were supplemented with (2-^13^C,^15^N)glycine, a peak appeared with *m*/*z* 121, consistent with formation of ^13^C/^15^N-doubly labeled NNG, validating Gly as an NNG precursor. Furthermore, *S. noursei* cultures fed both ^15^N/^13^C-Gly and l-(guanidino-^15^N_2_)arginine resulted in the appearance of a peak a with *m*/*z* of 122. This result suggested Arg as the nitro nitrogen donor.

**Fig 2:**
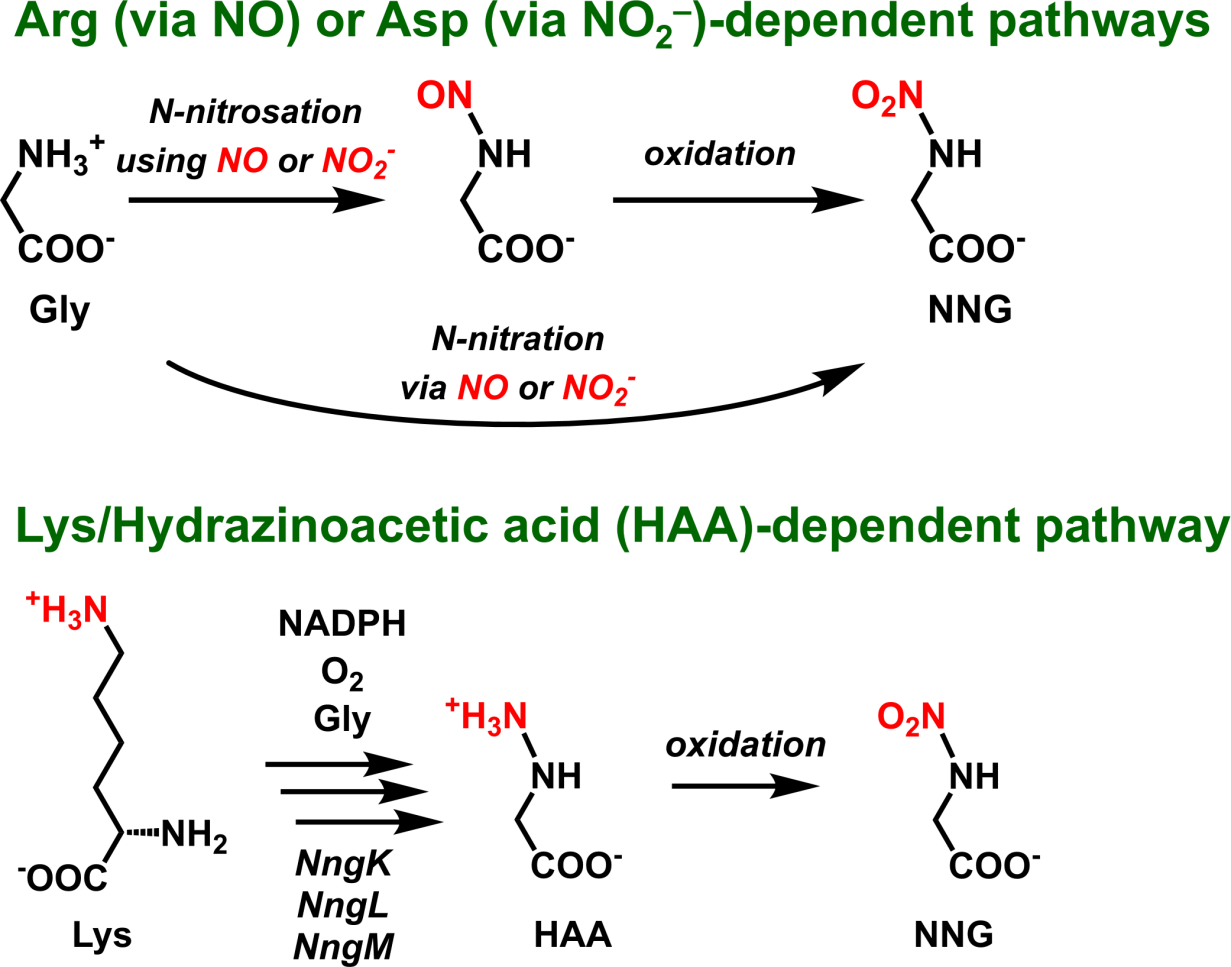
Proposed NNG biosynthesis pathways. The nitro group and its proposed origin in each proposed pathway are colored red.

One proposed pathway to explain this result is analogous to direct nitration of tryptophan (Trp) to 4-nitrotryptophan (9). *Streptomyces scabies* uses a bacterial nitric oxide (NO) synthase to oxidize Arg to form NO. This NO, along with dioxygen (O_2_), is a co-substrate in a cytochrome P450-mediated step that catalyzes the direct nitration of Trp to form 4-nitrotryptophan. One possible route to NNG is a direct *N*-nitration of Gly (Fig. 2, Arg/NO-dependent pathway). Alternatively, Ouazzani proposed a pathway by which activated NO *N*-nitrosates Gly to form *N*-nitrosoglycine, which is subsequently oxidized to form NNG (10). However, there is no clear NO source for *S. noursei,* as the bacterial genome lacks a bacterial NO synthase gene (8).

Therefore, we considered other hypotheses for NNG biosynthesis. *N*-nitrosation via nitrite (NO_2_^–^) is an established pathway in acidic conditions (11). In addition, NO_2_^–^-dependent *N*-nitrosation to form nitrosamine or diazo natural products has been observed in several biosynthetic pathways, including those for cremeomycin and triacsin (12–16). In these pathways, NO_2_^–^ is generated from the oxidation of aspartate by enzymes named CreE and CreD. Indeed, the *S. noursei* genome includes homologs of CreE and CreD (8). An alternate NO_2_^–^-dependent *N*-nitrosation pathway toward NNG can also be envisioned (Fig. 2, Asp/NO_2_^–^-dependent pathway) as an alternative to an NO-dependent pathway.

A third proposed NNG biosynthesis pathway occurs via *N*-oxygenation of the hydrazine natural product, hydrazinoacetic acid, HAA (Fig. 2, Lys/HAA-dependent pathway). A three-step biosynthetic pathway for the formation of HAA was discovered in *Streptomyces sp*. SoC090715LN-17 based on the Spb38, Spb39, and Spb40 enzymes (17). First, Spb38 hydroxylates Lys to N^6^-OH Lys. In the second step, Spb40 couples the N^6^-OH Lys with the α-amine of Gly to form the internal hydrazine *N*'-carboxymethylhydrazino-2-amino-hexanoic acid (CHAHA). Finally, Spb39 catalyzes oxidative cleavage of the CHAHA C–N bond to form l-2-aminoadipate 6-semialdehyde (AASA) and HAA. Several studies have reported the use of HAA or similar compounds in the biosynthesis of other N–N-bond-containing natural products (18–22). The *S. noursei* genome contains homologs for these *spb38-40* genes with conserved synteny (see below). This observation suggested that *S. noursei* has the enzymatic machinery to produce HAA. A pathway can be envisioned in which NNG results from *N*-oxygenation of HAA (Fig. 2, Lys/HAA-dependent pathway). Hereafter, these *S. noursei* homologs are referred to as *nngKLM*.

Furthermore, previously reported proteomics data reported by one of the authors indicate that these gene products exhibit increased abundance during NNG biosynthesis (8). Differential protein abundances of NngK, NngL, and NngM in stationary—when NNG is produced—vs. exponential growth phase cultures of *S. noursei* JCM-4701 are summarized in Table S1. The NngK protein from *S. noursei* (NCBI reference sequence WP_067356904.1) shares 58% amino acid identity with the Spb38 oxidase over 421 positions. The relative abundance of this protein was 9.8-fold higher in the stationary phase cells producing NNG compared to the exponential growth phase cells that did not produce NNG (*P* = 0.00835; one-way ANOVA; Table S1) (8). The NngL protein (WP_067356901.1) shares 55% amino acid identity with the Spg39 FAD-dependent oxidoreductase over 343 positions. The relative abundance of this protein was 210-fold higher in the stationary phase cells (*P* = 0.00019; ANOVA). The NngM protein (WP_067356904.1) shares 60% amino acid identity with the Spb40 ligase protein over the first 655 positions. The relative abundance of this protein was 480-fold higher in the stationary phase cells (*P* = 0.00001). These data further support the Lys/HAA pathway for NNG biosynthesis.

During the preparation of this manuscript, He and coworkers reported characterization of NNG biosynthesis by *Streptomyces yunnanensis* (7). Stable isotope studies in this report were consistent with the Lys-HAA-dependent pathway. Furthermore, the authors presented *in vitro* reconstitution data and gene knockout studies, providing insight into NNG biosynthesis. Their results indicated that HAA was oxidized to the corresponding hydrazone that was subsequently condensed onto the β-hydroxyl of serine via a non-ribosomal peptide synthase (NRPS). Gene knockout studies reported by the authors constrained the borders of the NNG biosynthetic gene cluster and identified essential genes for NNG biosynthesis. Their results are discussed in further detail below. Notably, the enzymes required to complete the oxidation to NNG remain unresolved.

To differentiate between the three hypotheses discussed above, we report stable isotope culture incorporation results, including (2-^13^C)HAA, and *in vitro* reconstitution of HAA biosynthesis. Our results are consistent with the conclusion also presented by He and co-workers that HAA is an NNG precursor. Our work also contributes analysis of ^15^N-NNG by LC-MS-MS provides direct evidence that the nitro nitrogen of NNG originates from Lys. We constrained the boundaries of the NNG BGC borders using a combination of bioinformatics and previously reported differential proteomics data (8). Furthermore, we report that azaserine is produced in *S. noursei* cultures, linking the biosyntheses of these natural products. Based on this observation, we propose an alternative pathway for the oxidation of HAA to NNG that will be discussed alongside that proposed by He and co-workers below. Finally, we report single-crystal X-ray diffraction and thermogravimetric analysis of NNG, positioning NNG as a candidate for further investigation as an energetic material. The implications of this study for the sustainable production of energetic nitramines are discussed below.

## MATERIALS AND METHODS

### Synthesis of HAA

Protocols for the synthesis of natural abundance and ^13^C-HAA are detailed in the supplemental material.

### Bacterial cultures and isotope-feeding experiments

*S. noursei* 48240 (ATCC 11455) was obtained from ATCC. Bacteria were grown on a maltose yeast extract medium agar plate, and spores were harvested and inoculated in liquid GYMB medium (0.4% dextrose, 0.4% yeast extract, 1.0% malt extract, and 1.0% beef extract at a pH of 7.4). The inoculated culture was incubated at 30°C with shaking for 3 days. For isotope incorporation experiments, pellets were washed and resuspended into sterile 50 mM sodium phosphate buffer at pH 7.5. The resuspended cells were incubated at 30°C for 24 h. Afterward, ^15^N-glycine (^15^N-Gly), l-(ε-^15^N)lysine (^15^N-Lys), L-(α-^15^N)aspartic acid (^15^N-Asp), l-(ω-^15^N_2_)arginine (^15^N_2_-Arg), or (2-^13^C)HAA were added to the cultures to a concentration of 10 mM. Labeled isotopes were purchased from Sigma Aldrich or Cambridge Isotopes; (2-^13^C)HAA was synthesized from (1-^13^C)bromoacetic acid using the synthetic protocol for HAA described in the supplemental material. The supplemented cultures were incubated for 24 h at 30°C with shaking. Cells were pelleted, and the supernatant was filtered. The filtrate was extracted by treatment with 100 mg NaCl/mL filtrate and 150 mg anhydrous MgSO_4_/mL filtrate. Afterward, the mixture was acidified with 17 µL 50% HCl/mL filtrate. Finally, acetonitrile was added at a ratio of 1 mL per mL of filtrate. The acetonitrile layer was collected, dried with nitrogen, and resuspended in 200 µL of ultrapure water prior to analysis.

### Recombinant expression, purification, and characterization of biosynthetic enzymes

The *nbtG*, *nngL*, and *nngM* genes were codon-optimized for *Escherichia coli* and synthesized (GenScript). The lysine *N*6-monooxygenase gene from *Nocardia farcinica* (*nbtG*) was cloned into the NdeI and XhoI cleavage sites of pET28-MBP-TEV. This expression vector encoded a gene product with an N-terminal maltose-binding protein domain and a TEV-protease cleavable linker. The *S. noursei nngL* and *nngM* genes were inserted in the NdeI and XhoI cleavage sites of pET-28a(+)-TEV. The resulting constructs were pET28-MBP-TEV-NbtG, pET-28a(+)-TEV-NngL, and pET-28a(+)-TEV-NngM. All constructs are expressed with an N-terminal hexahistidine tag.

The constructs pET28-MBP-TEV-NbtG and pET-28a(+)-TEV-NngL were transformed into chemically competent *E. coli* BL21(DE3) cells (New England Biolabs) via heat shock. Meanwhile, pET-28a(+)-TEV-NngM was transformed into *E. coli* BAP1 cells (Kerafast). Transformation mixtures were used to inoculate 50 mL of 2× YT medium containing 50 µg/mL kanamycin. These cultures were incubated overnight at 37°C with shaking. Afterward, 10 mL of the starter culture was used to inoculate 1 L of 2× YT/kanamycin or LB/kanamycin media. The inoculated media were incubated for 4–6 h at 37°C with shaking. At this time, the cultures were chilled at 4°C for 30 min, treated with 200 µM IPTG to induce expression, and incubated overnight at 18°C with shaking. Cells were harvested via centrifugation, and the pellets were immediately processed or stored at −60°C.

Cell pellets were resuspended in 20 mM Tris-HCl buffer with 150 mM NaCl at pH 8.0 (MBP-NbtG and NngM) or 50 mM 2-(Cyclohexylamino)ethanesulfonic acid (CHES) with 200 mM NaCl at pH 9.3 (NngL). For these latter preparations, 100 µM flavin adenine dinucleotide (FAD) and 25 µg/mL lysozyme were also added to the resuspension buffers and incubated with stirring for 30 min. The resuspended cells were lysed by sonication. The lysate was cleared by centrifugation, and the supernatant was collected. Proteins were purified using immobilized metal chelate affinity chromatography (IMAC) with Ni^2+^-HTC beads (GoldBio) by standard protocols. Purified protein was concentrated in a 30 kDa molecular weight cutoff Amicon centrifugal filter (Millipore), and the buffer was exchanged to an imidazole-free buffer. Glycerol was added to a final concentration of 5%, and the protein was aliquoted and stored at −60°C.

Protein concentration was determined by BCA assay (Pierce, Thermo Fisher). Protein-bound flavin was quantified by UV-vis absorption spectrophotometry using the flavin molar absorptivity coefficient of ε_450_ = 12,000 M^−1^cm^−1^. Zn^2+^ occupancy was determined using a colorimetric zinc assay kit (Abcam Limited).

### NNG crystallization

NNG was dissolved in anhydrous acetonitrile and filtered through 0.45 µm filters. Crystallization was achieved by slow evaporation of the filtered solution on a watch glass, resulting in thin colorless needles/threads within a week. The 0.128 × 0.037 × .019 mm crystal was mounted at −173°C.

### *In vitro* reconstitution

Testing of enzyme activities was performed by the following modified protocols reported for triacsin biosynthesis (23). Samples were prepared containing 1 mM Lys, 1 mM Gly, 2 mM NADH, 1 mM adenosine triphosphate (prepared in 200 mM Tris base at pH 8.0), and 100 µM MgCl_2_ in 50 mM 4-(2-hydroxyethyl)−1-piperazineethanesulfonic acid (HEPES) at pH 8. Enzymes were added to this mixture to a concentration of 20 µM to initiate the reaction. All samples were incubated in a heat block at 30°C for 2 h. Following incubation, two volumes of chilled acetonitrile buffered with 50 mM HEPES at pH 8 were added to each sample. The samples were then chilled at −20°C for 20 min. Afterward, the samples were centrifuged for 5 min, and 100 µL of each sample was combined with 25 mM of freshly prepared fluorenylmethyloxycarbonyl chloride (Fmoc-Cl) in −20°C acetonitrile to derivatize amine groups of amino acids and analytes. This derivatization reaction was incubated at room temperature for 10 min and subsequently filtered using 0.45 µm nylon membrane filters. Liquid chromatography coupled mass spectrometry (LC-MS) of the samples was performed immediately afterward.

### Analytical methods

Samples analyzed by liquid chromatography coupled time-of-flight mass spectrometry (LC-TOF-MS) were performed using an Agilent 1260 LC stack equipped with a Zorbax RX-C18 column (5 μm, 4.6 × 150 mm) connected to an Agilent 6230 TOF mass spectrometer with electrospray ionization (ESI). Separation of NNG in analytical samples was achieved by first pumping an isocratic solution of 10% methanol in water at 1 mL/min for 10 min. The column was washed between samples with a 5-min isocratic flush of 95% acetonitrile in water, followed by a 5-min flush with 10% methanol in water. Separation of derivatized CHAHA and HAA was achieved in a similar fashion, except that the isocratic 10% methanol eluent was run for 15 min to separate these analytes. The presence of NNG in samples was monitored in negative ion mode, while the presence of derivatized CHAHA and HAA was monitored in positive ion mode. The MS conditions use positive electrospray ionization. The drying gas ran at 350°C with a flow rate of 12 L/min. The nebulizer pressure was set to 40 psig. The capillary voltage was 3,750 V, with the fragmentor at 125 V and the skimmer at 65 V. The mass spectrometer was set with a capillary voltage of 3,500 V and a fragmentation voltage of 175 V. Commercially available synthetic NNG or HAA (AAblocks) were used as reference standards. Extracted ion chromatograms for the following analytes were generated using the listed masses: NNG ([M-H]^–^: *m*/*z* 119.01 ± 50 ppm), Fmoc-*N*^6^-OH-Lys ([M + H]^+^: *m*/*z* 358.18.24 ± 50 ppm), Fmoc-CHAHA ([M + H]^+^: 442.20 ± 50 ppm), and Fmoc_2_-HAA ([M + H]^+^: 535.1864 ± 20 ppm).

4′-phosphopantetheine incorporation in NngM was analyzed by a previously reported ejection method using LC-ESI-MS (24). Solvent A was 0.1% formic acid in water, and solvent B was acetonitrile, and the column used was an Agilent Poroshell 120 EC-C18 4 µm 2.1 x 100 mm. Two microliters of a 5 µM NngM sample were injected onto the column and eluted using the following conditions: 2 min 30% B, 10 min 30%–80% B, 3 min 95% B, and 3 min 30% B at 0.5 mL/min. Ejection conditions were set as 4,500 capillary voltage and 350 fragmentation voltage. During electrospray ionization, a fragment of the Ppant cofactor is ejected from the protein and can be observed on the extracted ion chromatogram with an *m*/*z* of 261.13 in positive ion mode.

The origin of the nitro nitrogen was determined using LC-MS-MS with a Xevo TQ-S Micro tandem quadrupole MS/MS coupled to an Acquity I-Class UPLC (Waters Corp.). Samples were injected (2 μL) on a Waters Acquity BEH C-18 (50 mm × 2.1 mm × 1.7 µm) column and eluted with a gradient of 30%–100% methanol in water (each containing 0.1% formic acid) at a flow rate of 0.400 mL/min over 3 min. Following each run, the instrument was flushed with 100% methanol for 0.75 min and equilibrated with 30% methanol for 2 min. The MS source was operated under ESI conditions, with a capillary voltage of 2,500 V and a cone voltage of 16 V. Acquisition of daughter ion spectra was performed using parent ion masses with *m*/*z* 119 Da and *m*/*z* 120 Da. Daughter ion scans were collected from *m*/*z* 40–125 Da using a collision energy of 9V.

Single-crystal X-ray diffraction was performed on a Bruker KAPPA PHOTON III DUO diffractometer equipped with an Oxford Cryostream 700. Data were collected using Mo Kα radiation from a molybdenum IμS DIAMOND microfocus tube. Data were collected in φ and ω scans and integrated using the Bruker Suite (25). The NNG crystal was found to be non-merohedrally twinned. Twinning was diagnosed, and the twin law was determined using the Domains utility in the Bruker Suite. Data were integrated using SAINT. Scaling and absorption correction were performed using SADABS, and the space group was determined using XPrep. The structure was solved using intrinsic phasing (26) and refined using the ShelX (26) package with Olex2 as a GUI (27).

Thermogravimetric analysis was performed on a Mettler Toledo TGA2 SF/XP1 equipped with a Pfeiffer ThermoStar mass spectrometer. The temperature was ramped at 10°C/min under a flow of dry helium.

## RESULTS AND DISCUSSION

### ^15^N/^13^C-Isotope labeling and origin of nitro nitrogen

**Fig 3:**
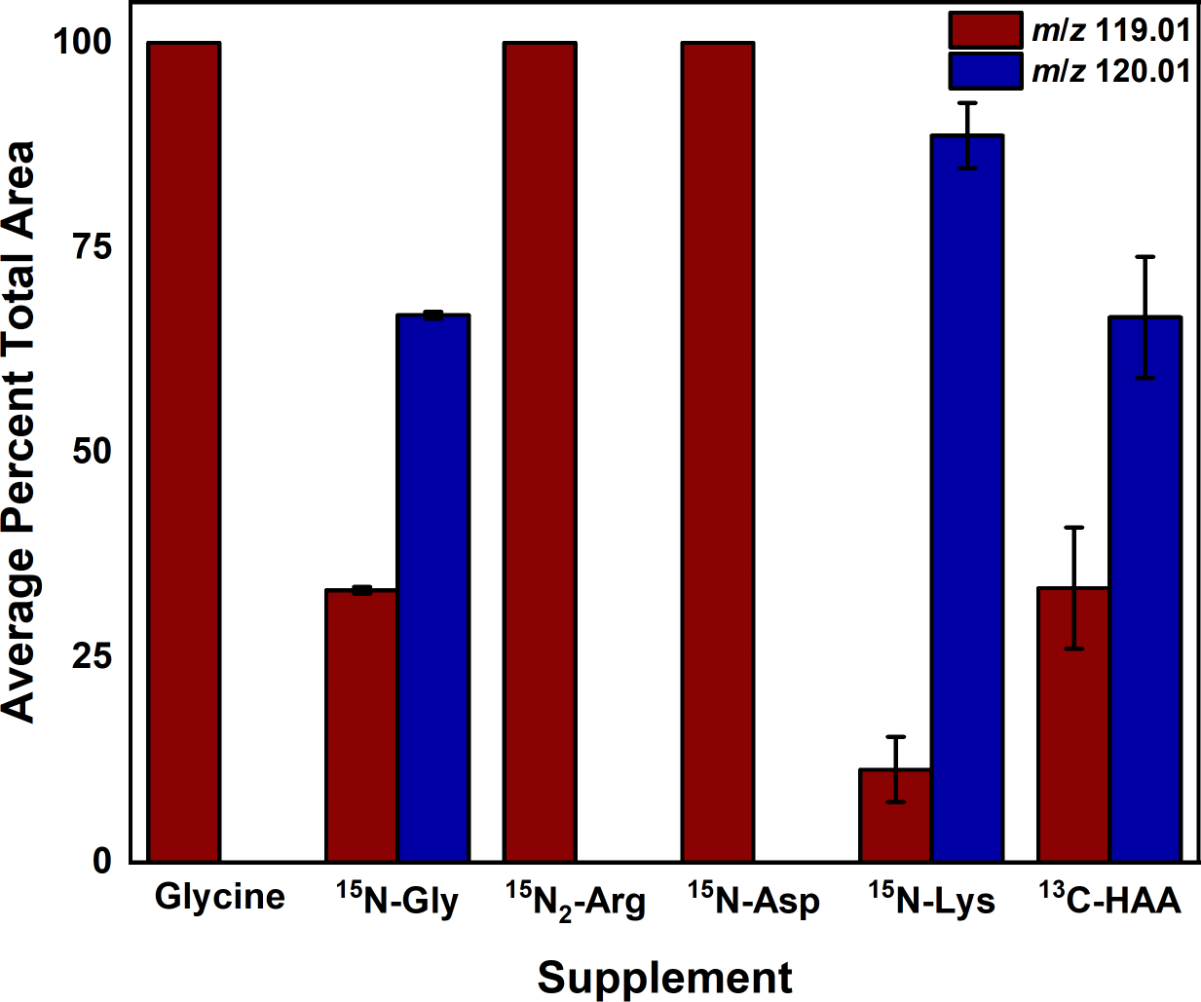
Incorporation of ^13^C or ^15^N stable isotopes into NNG. Cultures of *S. noursei* were supplemented with 10 mM of natural abundance Gly, ^15^N-Gly, ^15^N_2_-Arg, ^15^N-Asp, ^15^N-Lys, or ^13^C-HAA. LC-MS *m*/*z* 119 and 120 peaks were monitored, which are consistent with the [M–H]^–^ peak of unlabeled and ^15^N or ^13^C-labeled NNG, respectively. The bar heights show percent m/*z* 119 (red) or 120 (blue) vs. total NNG as determined from the extracted ion chromatogram (EIC) peak areas of the masses listed in the figure legend with ±50 ppm mass tolerance. Each bar shows the average of three replicates. The error bars represent one standard deviation. Representative mass spectra used to calculate these data are shown in Fig. S1.

Gly was previously shown to be a precursor for NNG and is presumed to donate the amino nitrogen of the nitramine moiety (8). To verify the previous results, *S. noursei* cells cultured in GYMB media were either supplemented with unlabeled glycine or ^15^N-glycine (^15^N-Gly). Total NNG was extracted from culture filtrates and analyzed by LC-TOF-MS. Controls lacking an amino acid supplement exhibited an extracted ion chromatogram peak at *m*/*z* 119 consistent with the [M–H]^–^ peak of NNG (Fig. S1A). The mass spectrum at this retention time also shows a peak at *m*/*z* 119 with no detection of a peak at *m*/*z* 120 (Fig. S1B). Meanwhile, an *m*/*z* 120 peak is apparent in filtrate extracts of *S. noursei* cultured with ^15^N-Gly (Fig. S1C). These averaged results are consistent with 67% ^15^N-Gly incorporation into NNG, which confirms the previously reported results that Gly is an NNG precursor (Fig. 3).

To differentiate between the three hypotheses for the origin of the nitro nitrogen discussed above (Arg, Asp, or Lys sources), *S. noursei* cells were mixed with l-(ω-^15^N_2_)arginine (^15^N_2_-Arg), l-(α-^15^N)aspartic acid (^15^N-Asp), or l-(ε-^15^N)lysine (^15^N-Lys). The results of these labeling experiments are summarized in Fig. 3. Filtrate extracts of *S. noursei* cultures supplemented with ^15^N-Arg or ^15^N-Asp showed no evidence for ^15^N-incorporation into NNG. However, cultures fed ^15^N-Lys resulted in the appearance of an *m*/*z* 120 peak with 88% incorporation (Fig. S1D). These results are consistent with the ε-N of Lys acting as the second nitrogen donor for NNG formation.

**Fig 4:**
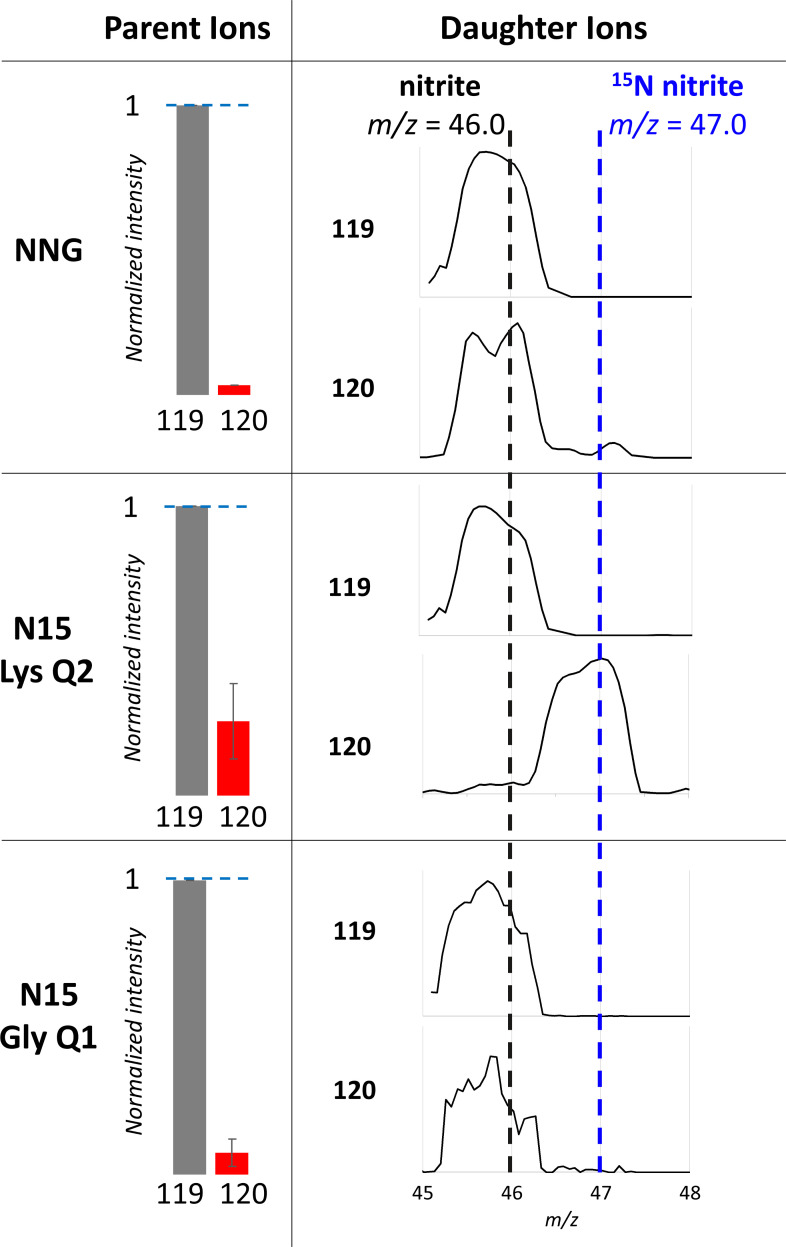
Identification of the biosynthetic origin of the nitro nitrogen of NNG. Left panels: Normalized intensities of [M−H]^−^ parent ions corresponding to NNG (*m/z* 119.0) and ^15^N-labeled NNG (*m*/*z* 120.0). Right panels: Mass spectra (*m*/*z* 45.0–48.0) obtained from the subsequent fragmentation of the *m*/*z* 119.0 and 120.0 parent ions for each sample. The daughter ions shown correspond to the molecular anion of NO_2_^–^ (*m*/*z* 46.0) or ^15^NO_2_^–^ (*m*/*z* 47.0).

To determine the origin of the NNG nitrogens, LC-MS-MS was performed on ^15^N-NNG samples prepared from either ^15^N-Gly or ^15^N-Lys labeling (Fig. 4). Fragmentation of the NNG parent ion at *m*/*z* 119 or 120 from a sample of unlabeled NNG resulted in a daughter ion at *m*/*z* 46. This peak is consistent with the [M]^−^ of NO_2_^–^, resulting from cleavage of the nitramine N–N bond. Fragmentation of the *m*/*z* 120 parent ion of ^15^N-NNG prepared ^15^N-Gly labeled samples exhibited the same *m*/*z* 46, consistent with unlabeled NO_2_^–^. Meanwhile, fragmentation of the *m*/*z* 120 parent ion of ^15^N-NNG prepared with ε-^15^N-Lys labeling resulted in a mass shift of the daughter ion to *m*/*z* 47, consistent with ^15^NO_2_^–^. These results showed that the nitrogen of the NNG nitro group originates from the ε-N of Lys and not the α-amine of Gly. The terminal nitrogen of HAA also originates from Lys. Therefore, these results support the hypothesis that HAA is a precursor of NNG.

To directly test this hypothesis, *S. noursei* cultures were supplemented with synthetic ^13^C-HAA. Analysis of these samples by LC-TOF-MS showed that a large increase in the *m*/*z* 120 peak, consistent with 66% incorporation of ^13^C-HAA into NNG (Fig. 3 and Fig. S1E). This result provides direct evidence that HAA is a precursor for NNG, supporting the proposed Lys/HAA-dependent pathway to NNG biosynthesis as shown in Fig. 2.

### Purification and characterization of HAA-producing proteins

To validate that NngK, NngL, and NngM produce HAA, we opted for an *in vitro* reconstitution approach; therefore, we required purified protein. Attempts to heterologously express soluble *S. noursei* NngK or a maltose-binding protein fusion (MBP-NngK) in *E. coli* failed. Therefore, we substituted *Nocardia farcinica* NbtG, as previously reported, as an N^6^-OH-Lys source for the study of pyrazomycin biosynthesis (28, 29). This protein (NCBI reference sequence BAD5560.1) exhibits 1% amino acid identity with NngK. It was purified as an *N*-terminal fusion with maltose-binding protein (MBP-NbtG) and an *N*-terminal hexahistidine tag, enabling purification by IMAC as previously reported (30). The SDS-PAGE analysis of the purified protein showed a protein band corresponding to an approximate mass of 90 kDa, which is consistent with the theoretical mass of MBP-NbtG of 86.8 kDa (Fig. S2A). The UV-vis absorption spectrum of purified protein is consistent with incorporation of a FAD cofactor into the protein (Fig. S3), which is consistent with previous reports (17, 23). Purified MBP-NbtG had 84% occupancy of FAD.

Isolated NngL was prepared by expression with an *N*-terminal hexahistidine tag in *E. coli* and purified by IMAC resin equilibrated with 50 mM CHES at pH 9.3. The high pH buffer was used to maintain the solubility of the protein due to its high isoelectric point (pI = 7.0). The SDS-PAGE of the purified protein showed a major band corresponding to an approximate mass of 40 kDa (Fig. S2B), which is consistent with the theoretical mass of 41.1 kDa. The UV-vis absorption spectrum of NngL is consistent with FAD binding to protein with an occupancy of 60% (Fig. S4).

Attempts to produce MBP-NngM in *E. coli* BL21 (DE3) or similar DE3 strains suffered from low yields of soluble protein. This typically manifested as samples of MBP-NngM with a large amount of contaminant proteins. Upon comparing the amino acid sequence of NngM to that of Spb40 from *Streptomyces sp.* SoC090715LN-17, we found that the Spb40 sequence exhibits only 87% coverage of the NngM sequence. Domain prediction showed the presence of a third predicted domain in the NngM sequence not observed in most homologs (Fig. S5), except the homolog required for azaserine biosynthesis. This third domain is annotated as an acyl carrier protein (ACP) domain with a 4′-phosphopantetheine (Ppant)-binding site. Therefore, the NngM protein (without the MBP fusion domain) was expressed in *E. coli* BAP1 cells, an engineered strain to biosynthesize and incorporate Ppant into compatible proteins (31). SDS-PAGE analysis of the proteins purified by IMAC using standard protocols showed a large protein band consistent with the 84.7 kDa theoretical mass of NngM (Fig. S6). Analysis of the Zn^2+^ occupancy showed the protein contained 1.0 Zn^2+^ per monomer protein. In addition, Ppant was detected using an ESI-LC-MS ejection assay, which directly detects a cyclized fragment of Ppant generated *in situ* and exhibits at a peak at *m*/*z* 261 (Fig. S7) (24).

### *In vitro* reconstitution of HAA biosynthesis

**Fig 5:**
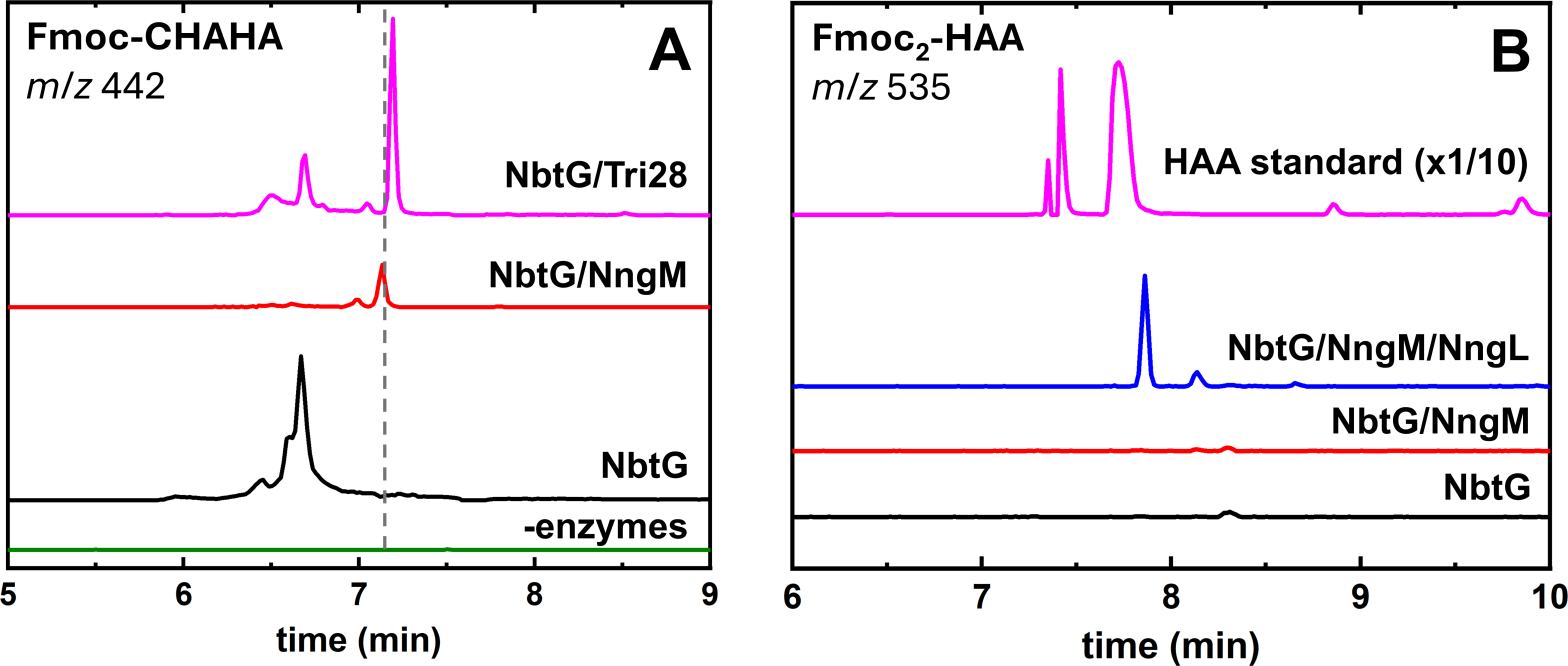
LC-MS extracted ion chromatograms (EICs) monitoring the appearance of Fmoc-derivatized CHAHA (**A**) or double Fmoc-derivatized HAA (**B**). Samples contain Lys, Gly, cofactors, and either NbtG (black trace), NbtG and NngM (red trace), or NbtG, NngM, and NngL (blue trace). In panel A, EICs of a negative control lacking any of the three enzymes (green trace) and a positive control for CHAHA that includes NbtG and Tri28 (magenta). In panel B, the positive control was an Fmoc-derivatized sample containing only synthetic HAA. Samples were prepared as discussed in the text. The dashed line shows the assigned retention time for Fmoc-derivatized CHAHA.

*In vitro* reconstitution of HAA biosynthesis using purified proteins was performed in a similar fashion, as previously reported (17–20). Reaction mixtures were prepared containing 1 mM Lys, 1 mM Gly, 2 mM NADH, 1 mM ATP, 100 µM MgCl_2_, 20 µM MBP-NbtG, and 20 µM *S. noursei* NngM in 50 mM HEPES at pH 8. Control samples containing all components except for *S. noursei* NngM were also prepared. All samples were incubated at 30°C for 2 h. After incubation, all samples were derivatized with Fmoc-Cl in acetonitrile. EICs were calculated from the LC-MS chromatograms monitoring the [M + H]^+^ ions of Fmoc-Lys (*m*/*z* 369), Fmoc-N^6^-OH-Lys (*m*/*z* 385), and Fmoc-CHAHA (*m*/*z* 442). All samples are consistent with enzymatic consumption of Lys based on comparison of the Fmoc-Lys signal intensity of the enzymatic reactions with a control containing only the substrates and cofactors. Addition of NbtG resulted in the appearance of Fmoc-derivatized N^6^-OH-Lys, which is consistent with the activity of NbtG (Fig. S8). All samples exhibited an intense peak for Fmoc-N^6^-OH-Lys. However, the hydroxylysine intermediate does not appear to be consumed even in reactions with NngM (data not shown). This is likely because NbtG and excess Lys remain in the samples, which replenishes any consumption of N^6^-OH-Lys. The samples were also monitored for Fmoc-CHAHA. Since synthetic CHAHA was not available, a positive control was generated by preparing reaction mixtures as described above with NngM replaced by Tri28—the enzyme responsible for N–N coupling of N^6^-OH-Lys and Gly to form CHAHA in triacsin biosynthesis (23). Both this positive control and samples containing *S. noursei* NngM exhibited peaks at 6.98 min that did not appear in samples lacking NngM (Fig. 5A), consistent with NngM-dependent CHAHA formation .

Next, we tested if *S. noursei* NngL converts CHAHA to HAA. Samples were prepared containing 1 mM Lys, 1 mM Gly, 2 mM NADH, 1 mM ATP, 100 μM MgCl_2_, and 20 µM each of MBP-NbtG, NngM, and NngL in 50 mM HEPES at pH 8.0. Controls lacking NngL or both of NngL and NngM were also prepared. EICs were calculated to monitor double-derivatized HAA (Fmoc_2_-HAA; *m*/*z* 535). Samples containing NbtG, NngM, and NngL exhibited a peak at 7.86 min that also appears in an Fmoc-derivatized HAA standard (Fig. 5B). Furthermore, this peak is absent in control samples lacking NngL. Therefore, this result is consistent with NngL-dependent conversion of CHAHA to HAA. The successful *in vitro* reconstitution of HAA biosynthesis from these enzymes indicates that *S. noursei* can produce HAA. Along with the data indicating that Lys, Gly, and HAA are incorporated into NNG, we conclude that *S. noursei* produces HAA as a precursor for NNG.

### Prediction of genes required for the oxidation of HAA to produce NNG

To identify candidate genes for NNG biosynthesis, we used antiSMASH to predict biosynthetic gene clusters (BGCs) within the *S. noursei* genome (32). By this analysis, we identified a 53.8 kbp BGC containing 43 genes, including *nngKLM*. This antiSMASH analysis also predicted a nearly identical BGC in the genome of *S. yunnanensis*, another known NNG-producing bacterium (Fig. S9) (7, 8).

**Fig 6:**
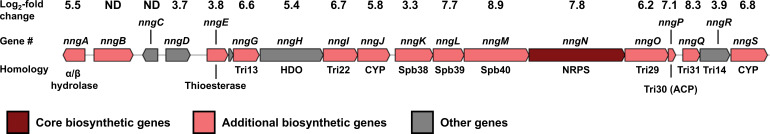
AntiSMASH predicted NNG biosynthetic gene cluster with borders suggested by differential proteomics data. Functional annotations (below cluster) based on amino acid sequence similarity with previously characterized biosynthesis enzymes involved in the installation of nitrogenous functional groups. Numbers on top show the log_2_-fold-change of the corresponding *S. noursei* gene product as reported previously Ref. 8 and shown in Table S1. Genes labeled ND indicate that the gene products were not detected in the proteomics data.

To constrain the borders of the NNG BGC, the AntiSMASH-predicted genes were cross-referenced with previously reported differential proteomics data (Table S1). The log_2_-fold increase of the corresponding gene products from the differential proteomics data suggests that only 17 of the 43 predicted genes are upregulated during NNG production. Sixteen of these upregulated genes are clustered together, suggesting the NNG BGC spans 19 predicted genes labeled *nngA* to *nngS* in Fig. 6. The AntiSMASH analysis also identified a binding site for AfsR—a *Streptomyces* antibiotic regulatory protein (SARP)—upstream of *orf15* (DUF4345) in Table S1, providing further evidence for the BGC boundaries. Three of these contiguous genes are not upregulated during NNG production, two of which are hypothetical proteins (*nngC* and *nngF*), and the third is annotated as an oxidoreductase (*nngB*). Therefore, the combined AntiSMASH and proteomics data constrain our consideration of the HAA oxidation pathway to 16 upregulated genes, three of which have already been assigned to HAA biosynthesis (*nngKLM*).

Six of the remaining 13 genes are annotated as enzymes also required for the biosynthesis of triacsin and azaserine—natural products that also require HAA as a precursor (18–20, 23). Indeed, the NNG BGC exhibits remarkably similar genetic inventory to the azaserine BGC of *Glycomyces harbinensis* (Fig. S10), suggesting the two biosynthetic pathways are similar. Several recent studies characterize azaserine biosynthesis. Each study uses different gene labels for the BGC components (18–20). For the following discussion, we will use the gene labels from Shikai et al., as it is the most recent and up-to-date work.

For azaserine biosynthesis, HAA biosynthesis is followed by succinylation of its hydrazine functional group by AzsS (Fig. S10). This succinyl-HAA is subsequently loaded by AzsP onto an acyl carrier protein (ACP), AzsQ, to form a succinyl-HAA-AzsQ adduct. An acyl-ACP dehydrogenase, AzsT, desuccinylates this intermediate to form the corresponding HAA adduct (HAA-AzsQ). A dehydrogenase, AzsF, oxidizes HAA-AzsQ to the 2-hydrazineylideneacetic acid (2-HYAA) adduct (HYAA-AzsQ). Shikai et al. reported that AzsN—the enzyme required for CHAHA formation en route to HAA biosynthesis—contains a C-terminal ACP domain (18). These authors showed that a 3-oxoacyl-ACP synthase, AzsD, could transfer 2-HYAA from HYAA-AzsQ to the truncated ACP domain of AzsN, forming an HYAA-AzsN-ACP adduct. The next step, forming a Ser-HYAA precursor dipeptide of azaserine, was shown to require the NRPS, AzsO. Addition of holo-AzsO with the truncated peptidyl carrier protein (PCP) domain of AzsO primed with Ser (Ser-AzsO-PCP) resulted in transfer and condensation of HYAA from HYAA-AzsN-ACP to the β-hydroxide group to form a HYAA-Ser-AzsO-PCP dipeptide adduct. Finally, evidence that a thioesterase, AzsB, hydrolyzes the HYAA-Ser dipeptide from AzsO was shown.

**Fig 7:**
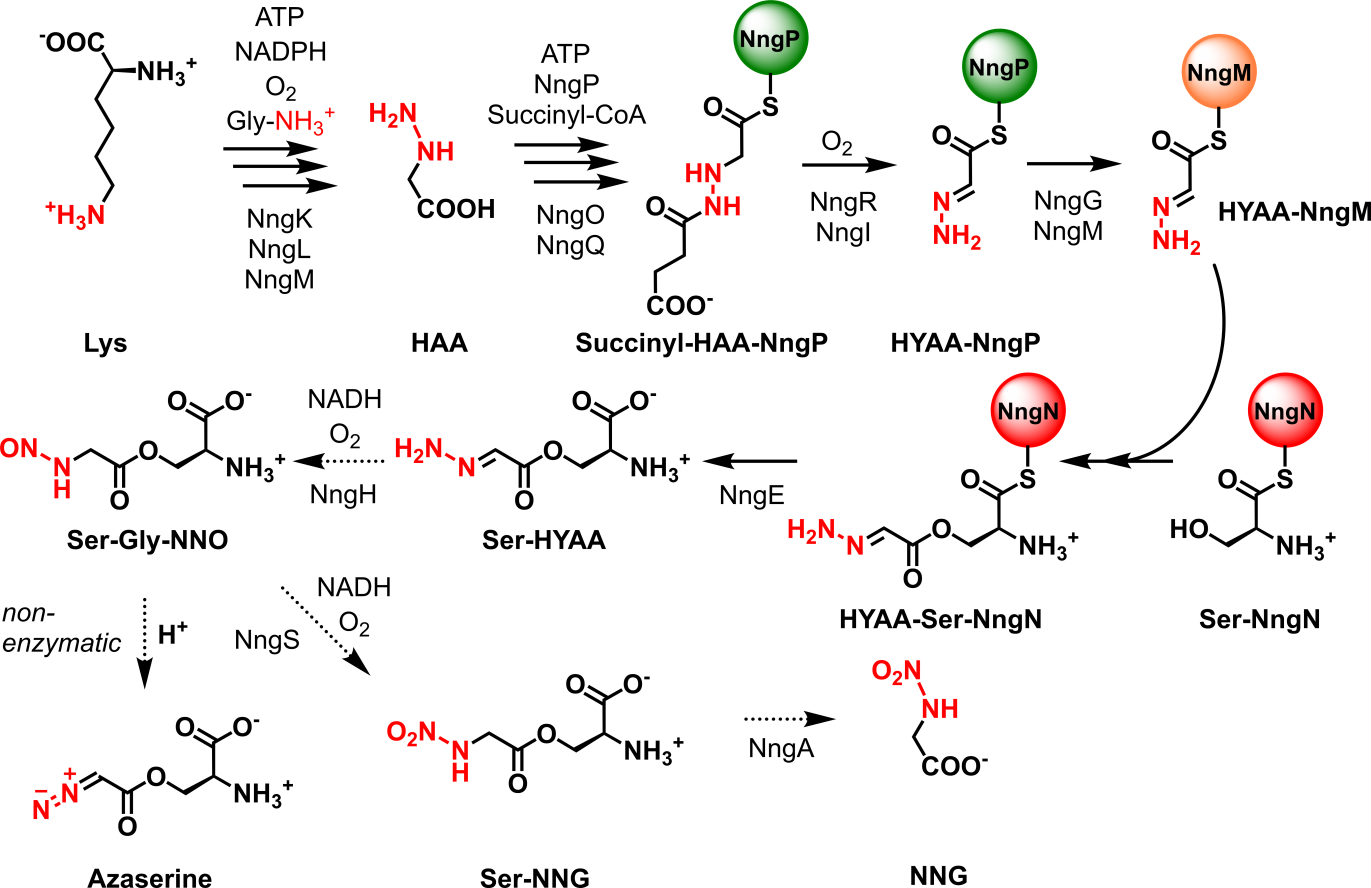
NNG biosynthetic pathway. Proposed *N*-oxidation steps that have not been biochemically validated herein or in reference 7 are shown in dashed arrows.

The recently published work by He and co-workers on NNG biosynthesis by *S. yunnanensis* showed that NNG biosynthesis follows that of azaserine biosynthesis up to formation of the Ser-HYAA dipeptide (7). These authors used gene knockout experiments to define the NNG BGC. In addition, they used an *in vitro* reconstitution approach to biochemically validate the activities of essential enzymes identified by the gene knockout experiments. A strategy similar to that used by Shikai et al. to study azaserine biosynthesis was used to show the formation of a Ser-HYAA adduct on a truncated PCP domain of the NRPS homolog, NngN. Treatment of this adduct with the thioesterase NngE resulted in its hydrolysis, but the Ser-HYAA cleavage product was not reported. Their results are consistent with the participation of *nngE*, *nngG*, *nngI*, *nngM*, *nngN, nngO*, *nngP*, *nngQ*, and *nngR*, all of which exhibit homology to azaserine biosynthetic genes (Fig. S10). In addition, the gene knockout experiments from this study showed that an α/β hydrolase, NngA, is necessary for NNG biosynthesis. The authors proposed that this protein hydrolyzes the Ser-NNG dipeptide to form NNG. These gene knockout studies also identify NngH and NngS as essential metalloenzymes, which the authors propose are essential for the oxidation of Ser-HYAA. The pathway resulting from this work is shown by the solid arrows shown in Fig. 7. Consistent with the work by He and co-workers, the differential proteomics show that each of these genes identified by the gene knockout studies is upregulated (Fig. 6). Thus, the BGC presented herein, built from a combination of bioinformatics and differential proteomics, is consistent with that defined by the gene knockout studies.

The oxidation pathway of Ser-HYAA to azaserine or NNG remains unresolved. A recent report from the Balskus group showed that a ferritin-like dioxygenase oxidizes similar hydrazone intermediates to the corresponding diazo products (22). However, a homolog of this protein is not found within our BGC, prompting consideration of other oxidation pathways by the five remaining unaccounted genes in Fig. 6 (*nngA*, *nngD*, *nngH*, *nngJ*, and *nngS*).

A heme dioxygenase-like (HDO) enzyme is one candidate to complete the oxidation of Ser-HYAA, the corresponding nitramine. An HDO homolog, NngH, was 42-fold more abundant in the stationary phase cells (*P* = 0.002). Previously reported HDO enzymes have non-heme diiron or iron-manganese binuclear active sites and catalyze *N*-oxygenase or *N*-hydroxylation activities (33–36). For example, the HDOs AznD and RohS catalyze 6-electron oxidation of 2-aminoimidazole to azomycin (8, 37). Meanwhile, the HDO domain of SznF catalyzes the 4-electron dihydroxylation of *N*-methylarginine (38, 39). Given the literature precedent of HDO domains to perform oxidations of nitrogenous functional groups in natural product biosynthetic pathways, it is expected that NngH participates in the oxidation of Ser-HYAA to Ser-NNG.

However, it is expected that a second metalloenzyme is also involved in the oxidation pathway. The NNG BGC in Fig. 6 includes two other genes capable of *N*-oxidation chemistry, *nngJ* and *nngS,* which both encode cytochromes P450 (CYPs). The NngJ and NngS proteins were 56-fold (*P* = 0.00017) and 110-fold more abundant in the stationary phase *S. noursei* cells compared to exponential growth phase cells that lacked NNG. A common function of CYPs is to hydroxylate substrates; furthermore, CYPs have been reported to exhibit *N*-hydroxylation activity (40–42). Thus, either of these enzymes has the potential to participate in the oxidation of Ser-HYAA to Ser-NNG. Notably, the gene knockout studies by He and co-workers clearly show that *nngS*, not *nngJ*, is required for NNG biosynthesis (7).

The azaserine BGC includes genes coding for an HDO (*azsE*) and only one CYP. The high homology between the azaserine and NNG BGCs suggests *azsE* corresponds to the *nngJ* and, therefore, lacks an *nngS* homolog (Fig. S10). Indeed, *G. harbinensis* AzsE (WP_143014975.1) shares 51.5% amino acid identity with NngJ but less than 23% with NngJ, further supporting homology of AzsE with NngS. As discussed above, *nngS*, not *nngJ*, is critical for NNG biosynthesis. Therefore, we propose that the presence of NngS catalyzes the oxidation of a common intermediate between the azaserine and NNG biosynthesis pathways that accounts for the formation of a nitramine moiety in the latter. By this proposed pathway, an HDO (NngE for NNG and AzsE for azaserine) is functionally equivalent, catalyzing hydroxylation of the terminal nitrogen of the HYAA-Ser dipeptide. The resulting hydroxylated product tautomerizes to the corresponding nitrosamine, as shown in Fig. S11. Bifurcation to form azaserine or the nitramine product is proposed to occur via this common nitrosated species (Ser-Gly-NNO) or the corresponding oxime tautomer. Acid-catalyzed degradation of primary nitrosamines to the diazo functionality is well established (43); therefore, Ser-Gly-NNO should non-enzymatically convert to azaserine. However, we propose that the presence of NngS in *S. noursei* rapidly catalyzes the oxidation of Ser-Gly-NNO to form the corresponding nitramine dipeptide (Ser-NNG). Supporting this proposed pathway, we did not observe NNG in culture extracts of *G. harbinensis* (data not shown). However, azaserine was observed in culture extracts of *S. noursei* (Fig. S12). The observation of azaserine alongside NNG in *S. noursei* cultures suggests that some Ser-Gly-NNO eludes oxidation by NngS, resulting, instead, in the non-enzymatic formation of azaserine (Fig. 7).

### Future prospects for the sustainable production of energetic nitramine materials

This study has shown that the hydrazine HAA is a precursor for NNG. Using NNG-producing bacteria or their purified enzymes can be envisioned to enable synthetic biology or biocatalytic approaches to produce high-energy nitramines. However, hydrazines are quite reactive in the biological milieu. Anammox bacteria exhibit a unique bioenergetics pathway, oxidizing NO to dinitrogen (N_2_) via hydrazine (N_2_H_4_) as an intermediate (44). These bacteria produce unique membrane-bound compartments called anammoxosomes that compartmentalize these reactions for energy conservation. In addition, this compartmentalization likely prevents exposure of the cell to N_2_H_4_, which is toxic to other bacteria (45). In the absence of such compartmentalization, a biocatalytic approach may prove more feasible than a synthetic biology approach. Regardless, identifying pathway machinery with substrate promiscuity to accommodate non-native hydrazines will be required first. An expected key enzyme in HAA oxidation is NngH. There are over 1,000 homologs of this protein in the database, suggesting that there are ample opportunities to identify biocatalysts with sufficient substrate promiscuity.

**Fig 8:**
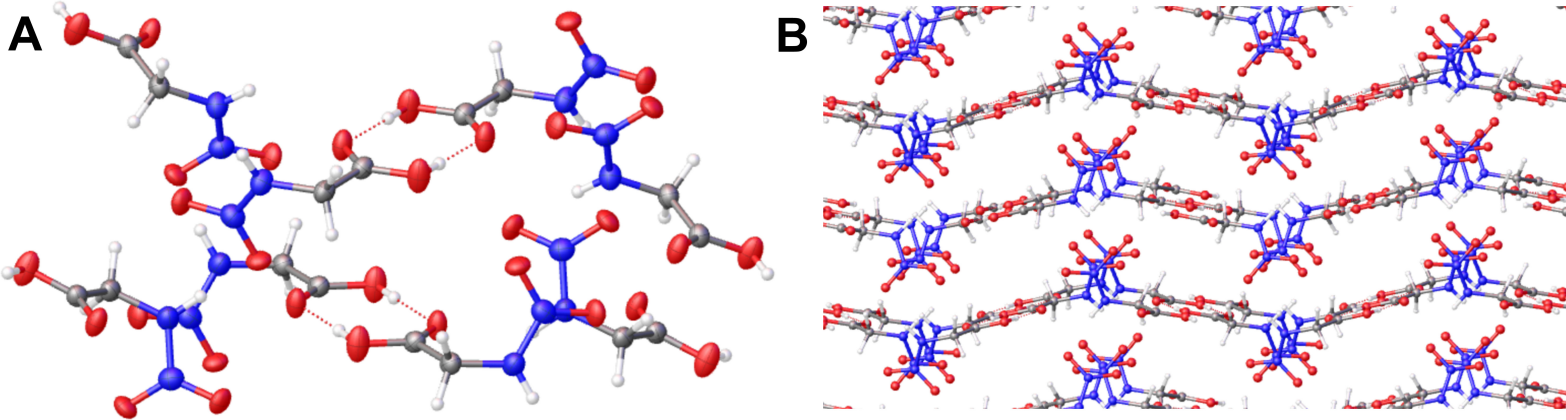
Crystal structure of NNG. (**A**) Thermal ellipsoid plot with ellipsoids set at 50% probability level, and hydrogen atoms represented by white spheres. Red, blue, and gray represent oxygen, nitrogen, and carbon, respectively. An aggregate of eight molecules is shown, illustrating the hydrogen bond pairing through carboxylic acid groups. (**B**) Packed ball-and-stick structure illustrating herringbone sheet pattern.

In addition, it is appropriate to consider the potential of NNG as an energetic material. The oxygen balance for NNG is −20.2% for formation of CO_2_, which is comparable to that for RDX (−21.6%), suggesting that it may be susceptible to detonation initiation. We determined the crystal structure of NNG using single-crystal X-ray diffraction (SC-XRD; Fig. 8). The crystal exhibited non-merohedral twinning (i.e., multiple crystals) and was diagnosed as a 5-component twin, which was partially treated by integration of multiple domains simultaneously. While the final residual *R*_1_ remained somewhat elevated (9%), the quality of the structure is good with low residual electron density (max peak = 0.8 e^-^), good ellipsoid shape, and no disorder. The structure quality was sufficiently good to locate and refine amine and carboxylic acid hydrogen atoms, demonstrating that NNG crystallizes in its neutral amino acid tautomer. The structure crystallizes as stacks of 2D herringbone sheets of hydrogen-bonded networks with NNG bound to one another within the sheet through pairs of carboxylic acid groups. The sheets are held together through dipole-dipole interactions between the negatively charged nitro-oxygen atoms and the positively charged amine protons. Additionally, the structure shows that NNG crystallizes with one molecule in the asymmetric unit as a pure material, not a hydrate, suggesting a favorable energetic crystallization phase. The crystallographic density of NNG is 1.615 at the temperature of data collection of −130°C.

Thermogravimetric analysis (Fig. S13) shows that NNG is stable beyond 100°C and decomposes in the range of 120°C–160°C, during which time approximately half of the mass is lost (60%). From 160°C to 320°C, most of the remaining mass is lost. The analysis of the MS data (Fig. S14) suggests that the initial weight loss from 120°C to 160°C is in the form of N_2_, and not carbon monoxide (CO) based upon observation of *m*/*z* peaks at 14 and 28, but very little atomic oxygen at *m*/*z* of 16 and no carbon at *m*/*z* of 12. Dihydrogen (H_2_) is also observed throughout the decomposition. Above 160°C, there is a small uptick in oxygen formation, suggesting deoxygenation of the remaining organic byproduct, but CO and CO_2_ are not observed. The result suggests that NNG does not decompose explosively via self-combustion under conditions of thermal decomposition. Rather, it decomposes largely into constituent elements, O_2_, N_2_, H_2_, and some nonvolatile byproducts, but an explosion does not occur. This suggests promising stability for use in energetic materials and that this material is not prone to explosive thermal decomposition, despite being well oxygen balanced for explosion.

### Conclusions

The combined results conclude that l-Lys, Gly, and HAA are precursors for NNG biosynthesis, with the nitro nitrogen being derived from the ε-N of l-Lys. An HAA-dependent pathway is supported by *in vitro* reconstitution of HAA biosynthesis using MBP-NbtG, NngL, and NngM. The BGC was defined by cross-referencing differential proteomics data with an antiSMASH-predicted BGC from the *S. noursei* genome. These data validate some of the conclusions of the recent NNG biosynthetic study (7). In addition to validating the origin of the NNG nitro nitrogen, we show that azaserine is produced alongside NNG in *S. noursei* cultures. This observation suggests that both azaserine and NNG biosynthesis share a common nitrosamine intermediate, Ser-Gly-NNO, from which non-enzymatic decomposition leads to azaserine, while its enzymatic oxidation results in the formation of the nitramine analog, Ser-NNG. Finally, the combined oxygen balance, NNG crystal structure, and TGA analysis of NNG prompt further investigation of NNG as a non-sensitive energetic material. Further investigation and development of this NNG biosynthetic pathway to oxidize non-native hydrazines or investigation of NNG, itself, as an energetic material could lead to the mitigation of waste produced during the manufacturing of energetic nitramines.

## Data Availability

Additional LC-MS chromatograms, mass spectra of culture filtrates and *in vitro* assays, characterization of purified proteins, synthetic protocols for HAA, and characterization of synthetic intermediates are included in the supplemental material. The crystallographic information file is available as File S1 and from the Cambridge Crystallographic Data Centre as deposition number 2531241. Crystallographic tables and TGA-MS data are available in File S2.

## SUPPLEMENTAL MATERIAL

File S1 aem.00631-26-s0001.cif Crystallographic information.

File S2 aem.00631-26-s0002.pdf Crystallographic tables and TGA-MS data.

Supplemental material aem.00631-26-s0003.docx Supplemental methods, Fig. S1 to S14, and Tables S1 to S8.

ASM does not own the copyrights to Supplemental Material that may be linked to, or accessed through, an article. The authors have granted ASM a non-exclusive, world-wide license to publish the Supplemental Material files. Please contact the corresponding author directly for reuse.

## References

[1] Agrawal JP. 2010. High energy materials: propellants, explosives and pyrotechnics. John Wiley & Sons.

[2] Abadin H, Smith C, Ingerman L, Llados FT, Barber LE, Plewak D, Diamond GL. 2013. Toxicological profile for RDX 24006555

[3] Song L-R, Fan Z, Zhang A. 2019. Recent advances in transition metal-catalyzed C(sp ^2^ )–H nitration. Org Biomol Chem 17:1351–1361. doi:10.1039/C8OB02750A 30644943

[4] Sheldon RA, Woodley JM. 2018. Role of biocatalysis in sustainable chemistry. Chem Rev 118:801–838. doi:10.1021/acs.chemrev.7b00203 28876904

[5] Parry R, Nishino S, Spain J. 2011. Naturally-occurring nitro compounds. Nat Prod Rep 28:152–167. doi:10.1039/c0np00024h 21127810

[6] Miyazaki Y, Kono Y, Shimazu A, Takeuchi S, Yonehara H. 1968. Production of nitraminoacetic acid by Streptomyces noursei. J Antibiot 21:279–282. doi:10.7164/antibiotics.21.279 5671992

[7] Wang Y, Zhang J, Shi R, Zhou S, Chen S, Li R, Huang W, He HY. 2025. Nitramine formation via a cryptic non‐ribosomal peptide synthetase‐dependent strategy in N ‐nitroglycine biosynthesis. Angew Chem Int Ed 64:e202507866. doi:10.1002/anie.202507866 40366104

[8] Graham DE, Spain JC, Parry RJ, Hettich RL, Mahan KM, Klingeman DM, Giannone RJ, Gulvick CA, Fida TT. 2018. Nitration enzyme toolkit for the biosynthesis of energetic materials (ORNL/SPR-2017/498). Oak Ridge National Lab, Oak Ridge, TN

[9] Barry SM, Kers JA, Johnson EG, Song L, Aston PR, Patel B, Krasnoff SB, Crane BR, Gibson DM, Loria R, Challis GL. 2012. Cytochrome P450–catalyzed L-tryptophan nitration in thaxtomin phytotoxin biosynthesis. Nat Chem Biol 8:814–816. doi:10.1038/nchembio.1048 22941045 PMC3522571

[10] Le Goff G, Ouazzani J. 2014. Natural hydrazine-containing compounds: biosynthesis, isolation, biological activities and synthesis. Bioorganic & Medicinal Chemistry 22:6529–6544. doi:10.1016/j.bmc.2014.10.011 25456382

[11] Hughes MN. 2008. Chemistry of nitric oxide and related species. Methods Enzymol 436:3–19. doi:10.1016/S0076-6879(08)36001-7 18237624

[12] Del Rio Flores A, Zhai R, Kastner DW, Seshadri K, Yang S, De Matias K, Shen Y, Cai W, Narayanamoorthy M, Do NB, Xue Z, Marzooqi DA, Kulik HJ, Zhang W. 2024. Enzymatic synthesis of azide by a promiscuous N-nitrosylase. Nat Chem 16:2066–2075. doi:10.1038/s41557-024-01646-2 39333393 PMC11611683

[13] Sugai Y, Katsuyama Y, Ohnishi Y. 2016. A nitrous acid biosynthetic pathway for diazo group formation in bacteria. Nat Chem Biol 12:73–75. doi:10.1038/nchembio.1991 26689788

[14] Waldman AJ, Balskus EP. 2018. Discovery of a diazo-forming enzyme in cremeomycin biosynthesis. J Org Chem 83:7539–7546. doi:10.1021/acs.joc.8b00367 29771512 PMC6425738

[15] Twigg FF, Cai W, Huang W, Liu J, Sato M, Perez TJ, Geng J, Dror MJ, Montanez I, Tong TL, Lee H, Zhang W. 2019. Identifying the biosynthetic gene cluster for triacsins with an N-hydroxytriazene moiety. Chembiochem 20:1145–1149. doi:10.1002/cbic.201800762 30589194 PMC6590916

[16] Wang M, Wei Z-W, Ryan KS. 2025. A heme-dependent enzyme forms the hydrazine in the antibiotic negamycin. Nat Chem Biol 21:1012–1020. doi:10.1038/s41589-025-01898-0 40312596

[17] Matsuda K, Tomita T, Shin-Ya K, Wakimoto T, Kuzuyama T, Nishiyama M. 2018. Discovery of unprecedented hydrazine-forming machinery in bacteria. J Am Chem Soc 140:9083–9086. doi:10.1021/jacs.8b05354 30001119

[18] Shikai Y, Kawai S, Katsuyama Y, Ohnishi Y. 2023. In vitro characterization of nonribosomal peptide synthetase-dependent O-(2-hydrazineylideneacetyl)serine synthesis indicates a stepwise oxidation strategy to generate the α-diazo ester moiety of azaserine. Chem Sci 14:8766–8776. doi:10.1039/d3sc01906c 37621439 PMC10445470

[19] Van Cura D, Ng TL, Huang J, Hager H, Hartwig JF, Keasling JD, Balskus EP. 2023. Discovery of the Azaserine biosynthetic pathway uncovers a biological route for α‐diazoester production. Angew Chem Int Ed 62:e202304646. doi:10.1002/anie.202304646 PMC10330308 37151182

[20] Wei Z-W, Niikura H, Wang M, Ryan KS. 2023. Identification of the Azaserine biosynthetic gene cluster implicates hydrazine as an intermediate to the diazo moiety. Org Lett 25:4061–4065. doi:10.1021/acs.orglett.3c01229 37235858

[21] Shi J, Zang X, Zhao Z, Shen Z, Li W, Zhao G, Zhou J, Du Y-L. 2023. Conserved enzymatic cascade for bacterial azoxy biosynthesis. J Am Chem Soc 145:27131–27139. doi:10.1021/jacs.3c12018 38018127

[22] Pfeifer K, Van Cura D, Wu KJY, Balskus EP. 2026. Chemical capture of diazo metabolites reveals biosynthetic hydrazone oxidation. Nature 652:517–525. doi:10.1038/s41586-025-10079-x 41639443 PMC13061610

[23] Del Rio Flores A, Twigg FF, Du Y, Cai W, Aguirre DQ, Sato M, Dror MJ, Narayanamoorthy M, Geng J, Zill NA, Zhai R, Zhang W. 2021. Biosynthesis of triacsin featuring an N-hydroxytriazene pharmacophore. Nat Chem Biol 17:1305–1313. doi:10.1038/s41589-021-00895-3 34725510 PMC8605994

[24] Meluzzi D, Zheng WH, Hensler M, Nizet V, Dorrestein PC. 2008. Top-down mass spectrometry on low-resolution instruments: characterization of phosphopantetheinylated carrier domains in polyketide and non-ribosomal biosynthetic pathways. Bioorganic & Medicinal Chemistry Letters 18:3107–3111. doi:10.1016/j.bmcl.2007.10.104 18006314 PMC2519147

[25] Bruker S, SAINT S. 2002. Bruker AXS Inc. Madison, Wisconsin, USA.

[26] Sheldrick GM. 2015. SHELXT - integrated space-group and crystal-structure determination. Acta Crystallogr A Found Adv 71:3–8. doi:10.1107/S2053273314026370 25537383 PMC4283466

[27] Dolomanov OV, Bourhis LJ, Gildea RJ, Howard JAK, Puschmann H. 2009. OLEX2: a complete structure solution, refinement and analysis program. J Appl Crystallogr 42:339–341. doi:10.1107/S0021889808042726

[28] Zhao G, Yao S, Rothchild KW, Liu T, Liu Y, Lian J, He HY, Ryan KS, Du YL. 2020. The biosynthetic gene cluster of pyrazomycin-A C-nucleoside antibiotic with a rare pyrazole moiety. Chembiochem 21:644–649. doi:10.1002/cbic.201900449 31482654

[29] Zhao G, Peng W, Song K, Shi J, Lu X, Wang B, Du Y-L. 2021. Molecular basis of enzymatic nitrogen-nitrogen formation by a family of zinc-binding cupin enzymes. Nat Commun 12:7205. doi:10.1038/s41467-021-27523-x 34893622 PMC8664883

[30] Binda C, Robinson RM, Martin Del Campo JS, Keul ND, Rodriguez PJ, Robinson HH, Mattevi A, Sobrado P. 2015. An unprecedented NADPH domain conformation in lysine monooxygenase NbtG provides insights into uncoupling of oxygen consumption from substrate hydroxylation. J Biol Chem 290:12676–12688. doi:10.1074/jbc.M114.629485 25802330 PMC4432286

[31] Pfeifer BA, Admiraal SJ, Gramajo H, Cane DE, Khosla C. 2001. Biosynthesis of complex polyketides in a metabolically engineered strain of E. coli. Science 291:1790–1792. doi:10.1126/science.1058092 11230695

[32] Blin K, Shaw S, Vader L, Szenei J, Reitz ZL, Augustijn HE, Cediel-Becerra JDD, de Crécy-Lagard V, Koetsier RA, Williams SE, et al.. 2025. antiSMASH 8.0: extended gene cluster detection capabilities and analyses of chemistry, enzymology, and regulation. Nucleic Acids Res 53:W32–W38. doi:10.1093/nar/gkaf334 40276974 PMC12230676

[33] Powell MM, Rao G, Britt RD, Rittle J. 2023. Enzymatic hydroxylation of aliphatic C-H bonds by a Mn/Fe cofactor. J Am Chem Soc 145:16526–16537. doi:10.1021/jacs.3c03419 37471626 PMC10401708

[34] Manley OM, Phan HN, Stewart AK, Mosley DA, Xue S, Cha L, Bai H, Lightfoot VC, Rucker PA, Collins L, Williams TI, Chang W-C, Guo Y, Makris TM. 2022. Self-sacrificial tyrosine cleavage by an Fe:Mn oxygenase for the biosynthesis of para-aminobenzoate in Chlamydia trachomatis. Proc Natl Acad Sci U S A 119:e2210908119. doi:10.1073/pnas.2210908119 36122239 PMC9522330

[35] Andersson CS, Högbom M. 2009. A Mycobacterium tuberculosis ligand-binding Mn/Fe protein reveals a new cofactor in a remodeled R2-protein scaffold. Proc Natl Acad Sci USA 106:5633–5638. doi:10.1073/pnas.0812971106 19321420 PMC2667070

[36] McBride MJ, Pope SR, Hu K, Okafor CD, Balskus EP, Bollinger JM, Boal AK. 2021. Structure and assembly of the diiron cofactor in the heme-oxygenase-like domain of the N-nitrosourea-producing enzyme SznF. Proc Natl Acad Sci USA 118:118 (doi:10.1073/pnas.2015931118 PMC7848743 33468680

[37] Hedges JB, Ryan KS. 2019. In vitro reconstitution of the biosynthetic pathway to the nitroimidazole antibiotic azomycin. Angew Chem Weinheim Bergstr Ger 131:11773–11777. doi:10.1002/ange.201903500 31231913

[38] He H-Y, Henderson AC, Du Y-L, Ryan KS. 2019. Two-enzyme pathway links l-arginine to nitric oxide in N-nitroso biosynthesis. J Am Chem Soc 141:4026–4033. doi:10.1021/jacs.8b13049 30763082

[39] Ng TL, Rohac R, Mitchell AJ, Boal AK, Balskus EP. 2019. An N-nitrosating metalloenzyme constructs the pharmacophore of streptozotocin. Nature 566:94–99. doi:10.1038/s41586-019-0894-z 30728519 PMC6369591

[40] Seger ST, Rydberg P, Olsen L. 2015. Mechanism of the N-hydroxylation of primary and secondary amines by cytochrome P450. Chem Res Toxicol 28:597–603. doi:10.1021/tx500371a 25651340

[41] Ji L, Schüürmann G. 2013. Model and mechanism: N-hydroxylation of primary aromatic amines by cytochrome P450. Angew Chem Int Ed 52:744–748. doi:10.1002/anie.201204116 23169575

[42] Guo H, Bai X, Yang Q, Xue Y, Chen D, Tao J, Liu W. 2021. NocU is a cytochrome P450 oxygenase catalyzing N -hydroxylation of the indolic moiety during the maturation of the thiopeptide antibiotics nocathiacins. Org Biomol Chem 19:8338–8342. doi:10.1039/D1OB01284C 34523664

[43] Beard JC, Swager TM. 2021. An organic chemist’s guide to N-nitrosamines: their structure, reactivity, and role as contaminants. J Org Chem 86:2037–2057. doi:10.1021/acs.joc.0c02774 33474939 PMC7885798

[44] Kartal B, de Almeida NM, Maalcke WJ, Op den Camp HJM, Jetten MSM, Keltjens JT. 2013. How to make a living from anaerobic ammonium oxidation. FEMS Microbiol Rev 37:428–461. doi:10.1111/1574-6976.12014 23210799

[45] Kane DA, Williamson KJ. 1983. Bacterial toxicity and metabolism of hydrazine fuels. Arch Environ Contam Toxicol 12:447–453. doi:10.1007/BF01057588

