# Supplementary material for: Hydrazinoacetic acid is a biosynthetic precursor of the bacterially produced nitramine, N-nitroglycine
Source: Appl Environ Microbiol. 2026 May 20;92(6):e00631-26. doi: 10.1128/aem.00631-26 (PMC13274452; doi:10.1128/aem.00631-26)
Supplement: Supplemental material — Supplemental methods, Fig. S1 to S14, and Tables S1 to S8. [file aem.00631-26-s0003.docx]

**Supporting Information for**

**Hydrazinoacetic acid is a biosynthetic precursor of the bacterial nitramine, N-nitroglycine** Gabriel Padilla,^1,‡^ Benjamin M. Rathman, ^1,‡^ Shivaiah Vaddypally,^3^ Brenda Martinez Rodriguez, ^1^ Michael J. Zdilla,^3^ David E. Graham,^2^ Jonathan D. Caranto^1,*^

^1^Department of Chemistry, University of Central Florida, Orlando, FL 32816

^2^Biosciences Division, Oak Ridge National Laboratory, Oak Ridge, TN 37831

^3^Department of Chemistry, Temple University, Philadelphia, PA 19122

‡These authors contributed equally to this work

Corresponding Author: Jonathan D. Caranto ([jonathan.caranto@ucf.edu](mailto:jonathan.caranto@ucf.edu))

| **Table of contents** | |
| --- | --- |
| **General Synthetic Protocols** | pg. 3 |
| **Synthesis of BrAcOPP (1)** | pg. 4 |
| **Synthesis of ^13^C isotopically labeled BrAcOPP (4)** | pg. 4 |
| **Synthesis of H-(Nα-NHBoc)Gly-OPP (2)** | pg. 5 |
| **Synthesis of ^13^C isotopically labeled H-(Nα-NHBoc)Gly-OPP (5).** | pg. 5 |
| **Synthesis of hydrazinoacetic acid (3).** | pg. 5 |
| **Synthesis of ^13^C isotopically labeled hydrazinoacetic acid (6).** | pg. 6 |
| **Fig. S1.** Representative LC-MS data monitoring NNG produced during S. noursei culture feeding experiments. | pg. 7 |
| **Fig. S2.** SDS-PAGE of Ni^2+^-HTC purified MBP-NbtG and Nng39 | pg. 8 |
| **Fig. S3.** Uv-visible absorption spectrum of MBP-NbtG. | pg. 9 |
| **Fig. S4.** Uv-visible absorption spectrum of Nng39. | pg. 9 |
| **Fig. S5.** Comparison of predicted domain architecture for Spb40 vs. Nng40 based on the amino acid sequence. | pg. 10 |
| **Fig. S6.** SDS-PAGE of Ni^2+^-HTC purified Nng40 expressed in E. coli BAP1 cells | pg. 10 |
| **Fig. S7.** LC-MS extracted ion chromatograms monitoring PPant incorporation in purified Nng40. | pg. 11 |
| **Fig. S8.** Extracted ion chromatograms monitoring Fmoc-derivatized N^6^-OH-l-Lys | pg. 12 |
| **Fig. S9.** Comparison of antiSMASH-predicted BGCs in the genomes of the NNG-producing bacteria | pg. 12 |
| **Fig. S10.** Comparison of NNG and azaserine BGCs (top) and azaserine biosynthetic pathway. | pg. 13 |
| **Fig. S11.** Proposed bifurcation of a common nitrosamine intermediate to form both azaserine and NNG | Pg. 14 |
| **Fig. S12.** Detection of azaserine in culture extracts of *S. noursei.* | Pg. 15 |
| **Fig. S13.** TGA trace of a 3.3560 mg sample of NNG, showing three major mass loss regions | pg. 16 |
| **Fig. S14.** EI mass spectrum of evolved gasses during TGA analysis | pg. 16 |
| **Table S1.** AntiSMASH predicted BGC in S. noursei genome and annotations vs. experimental log_2_-fold differential expression of gene products | pg. 17 |
| **Table S2.** Crystal data and structure refinement for mo_NNG_twin_a. | pg. 21 |
| **Table S3.** Fractional Atomic Coordinates (×104) and Equivalent Isotropic Displacement Parameters (Å2×103) for mo_NNG_twin_a. | pg. 22 |
| **Table S4.** Anisotropic Displacement Parameters (Å2×103) for mo_NNG_twin_a. | pg. 22 |
| **Table S5.** Bond Lengths for mo_NNG_twin_a. | pg. 22 |
| **Table S6.** Bond Angles for mo_NNG_twin_a. | pg. 22 |
| **Table S7.** Torsion Angles for mo_NNG_twin_a. | pg. 23 |
| **Table S8.** Hydrogen Atom Coordinates (Å×104) and Isotropic Displacement Parameters  (Å2×103) for mo_NNG_twin_a. | pg. 23 |
| SC-XRD Experimental | pg. 23 |
| NMR characterization of HAA synthetic intermediates and products | pg. 24 |

***General Synthetic Procedures.*** Unless stated otherwise, reactions were performed in flame-dried glassware under a positive pressure of nitrogen gas using dry solvents. Commercial grade reagents and solvents were used without further purification except where noted. Other anhydrous solvents were purchased directly from chemical suppliers and used without further purification. Thin-layer chromatography (TLC) was performed using Silicycle F254 silica gel pre-coated aluminum-backed plates (0.2 mm). Flash chromatography was performed using silica from Silicycle (Siliaflash® P60, 40-63 µm, 230-400 mesh). Reaction progress was monitored by TLC analysis (single spot/two solvent systems) using a UV lamp, CAM (ceric ammonium molybdate), ninhydrin, or basic KMnO_4_ stain(s) for detection purposes. NMR spectra were recorded on a Bruker 400 MHz spectrometer. Proton chemical shifts are reported as δ values relative to residual signals from deuterated solvents (CDCl_3_ or D_2_O). HRMS of purified molecules were acquired using a Bruker Impact II ESI-QTOF.

**Synthesis of BrAcOPP (1).** Prepared according to previously reported procedure.^1^ In short, to NaH (60% dispersion in mineral oil, 32 mg, 0.79 mmol) in Et_2_O (1 mL) 2-phenyl-2-propanol (1081 mg, 7.938 mmol) was added dropwise as a solution in Et_2_O (3 mL) over 5 min. The reaction was allowed to stir for 20 min then cooled to -15°C. Trichloroacetonitrile (758 μL, 7.56 mmol) was added dropwise over 5 min and the reaction was allowed to warm to room temp over 1 h. The Et_2_O was then removed in vacuo and the resulting syrup was sonicated in hexanes (6 mL). The precipitate was filtered and washed with hexanes (2 x 6 mL) and the filtrate was concentrated in vacuo to afford a light-brown oil. To this oil, bromoacetic acid (525 mg, 3.78 mmol) was added as a solution in DCM (4.5 mL) and the reaction was stirred at room temp for 16 h. The precipitate was then filtered and washed with DCM (2 x 10 mL) and the filtrate was concentrated in vacuo. Purification by flash chromatography over silica gel (5-10% EtOAc:hexanes) afforded **1** as a colorless oil (967 mg, 3.76 mmol, 99%). Spectra matched those previously reported.^1^

**Synthesis of ^13^C isotopically labeled BrAcOPP (4).** Prepared in manner similar to **1**, **4** was obtained as a colorless oil (940 mg, 3.64 mmol, 96%). ^1^H NMR (400 MHz, CDCl_3_) δ 7.34-7.24 (m, 4H), 7.22-7.16 (m, 1H), 3.71 (d, J = 4.6 Hz, 1H), 1.73 (s, 6H); ^13^C{^1^H} NMR (101 MHz, CDCl_3_) δ 165.6, 144.9 (d, *J* = 1.3 Hz), 128.5, 127.5, 124.4, 84.0 (d, *J* = 2.8 Hz), 28.40 (d, *J* = 1.8 Hz), 27.29 (d, *J* = 64.9 Hz); HRMS (ESI-TOF) *m/z* [M + Na]^+^ calcd for C_10_^13^CH_13_BrO_2_Na^+^ 280.0025, found 280.0033.

**Synthesis of H-(Nα-NHBoc)Gly-OPP (2).** To a solution of *tert*-butyl carbazate (596 mg, 4.51 mmol) in DMSO (2.5 mL), NaHCO_3_ (948 mg, 11.28 mmol) was added and the reaction vigorously stirred. **1** (967 mg, 3.76 mmol) was added as a solution in DMSO (1.25 mL) over 10 min. The reaction continued to stir at room temp for 1.5 h. The reaction mixture was diluted with EtOAc (50 mL) and poured into H_2_O (50 mL). The organic layer was removed, and the aqueous layer was further extracted with EtOAc (2 x 50 mL). The organic layers were combined, washed with brine (50 mL), dried over anhydrous MgSO_4_, filtered, and concentrated. Purification by flash chromatography over silica gel (10-30% EtOAc:hexanes) afforded **2** as a white solid (995 mg, 3.23 mmol, 86%). ^1^H NMR (400 MHz, CDCl_3_) δ 7.38-7.30 (m, 4H), 7.29-7.22 (m, 1H), 6.36 (bs, 1H), 3.64 (s, 2H), 3.33 (bs, 1H), 1.79 (s, 6H), 1.45 (s, 9H); ^13^C{^1^H} NMR (101 MHz, CDCl_3_) δ 170.0, 156.2, 145.4, 128.5, 127.4, 124.3, 83.0, 80.7, 53.5, 28.7, 28.4; HRMS (ESI-TOF) *m/z* [M + H]^+^ calcd for C_16_H_25_N_2_O_4_^+^ 309.1809, found 309.1810.

**Synthesis of ^13^C isotopically labeled H-(Nα-NHBoc)Gly-OPP (5).** Prepared in manner similar to **2**, **5** was obtained as a white solid (991 mg, 3.20 mmol, 96%). ^1^H NMR (400 MHz, CDCl_3_) δ 7.36-7.30 (m, 4H), 7.28-7.22 (m, 1H), 6.38 (s, 1H), 3.63 (d, *J* = 5.5 Hz, 2H), 3.55 (bs, 1H), 1.79 (s, 6H), 1.45 (s, 9H); ^13^C{^1^H} NMR (101 MHz, CDCl_3_) δ 169.9, 156.1, 145.3, 128.4, 127.2, 124.2, 82.89 (d, *J* = 3.0 Hz), 80.5, 53.39 (d, *J* = 59.5 Hz), 28.63 (d, *J* = 1.8 Hz), 28.3; HRMS (ESI-TOF) *m/z* [M + H]^+^ calcd for C_15_^13^CH_25_N_2_O_4_^+^ 310.1842, found 310.1840.

**Synthesis of hydrazinoacetic acid (HAA, 3). 2** (960 mg, 3.11 mmol) was dissolved in a 1:1 mixture of TFA:DCM (8 mL) and stirred at rt for 2 h. The organic solvents were then removed in vacuo and the resulting residue was dissolved in 1M HCl (2 mL) and purified by SPE using a Hypersep C18 cartridge (1g) eluting with 1M HCl. Fractions containing product were combined and the solvent was removed in vacuo. The residue was dissolved in water and lyophilized to afford **HAA (3)** as the HCl salt, a yellowish-white solid (338 mg, 2.67 mmol, 86%). ^1^H NMR (400 MHz, D_2_O) δ 3.53 (s, 2H); ^13^C{^1^H} NMR (101 MHz, D_2_O) δ 171.6, 49.3; HRMS (ESI-TOF) *m/z* [M + H]^+^ calcd for C_2_H_7_N_2_O_2_^+^ 91.0502, found 91.0506.

**Synthesis of ^13^C isotopically labeled hydrazinoacetic acid (6).** Prepared in manner similar to **3**, **6** was obtained as the HCl salt, a white solid (214 mg, 1.68 mmol, 56%). ^1^H NMR (400 MHz, CDCl_3_) δ 3.84 (d, J = 5.4 Hz, 1H); ^13^C{^1^H} NMR (101 MHz, CDCl_3_) δ 172.0, 49.54 (d, J = 57.2 Hz); HRMS (ESI-TOF) *m/z* [M + H]^+^ calcd for C^13^CH_7_N_2_O_2_^+^ 92.0536, found 92.0539.


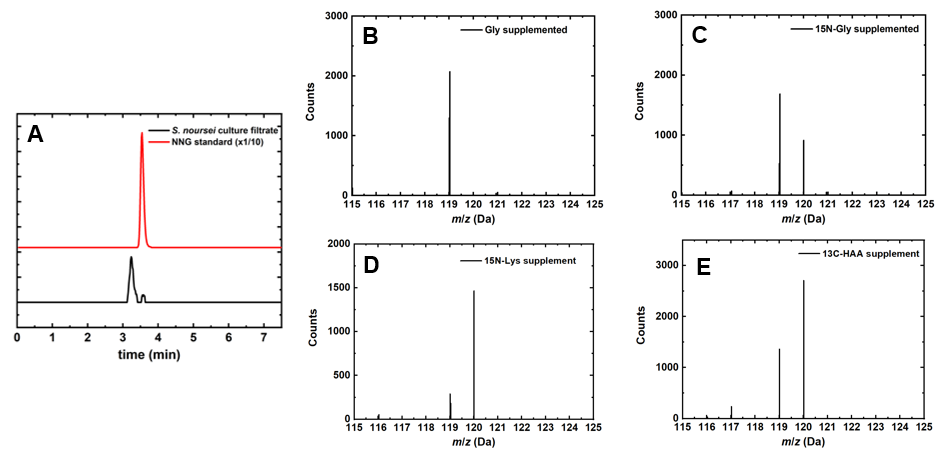


**Fig. S1**. Representative LC-MS data monitoring NNG produced during *S. noursei* culture feeding experiments. **A)** Representative extracted ion chromatogram (EIC) monitoring the [M-H]^–^ peak of NNG (*m*/*z* 119.01 ± 50 ppm) from *S. noursei* culture extracts compared with a synthetic NNG standard in 50 mM potassium phosphate buffer at pH = 8.0. Representative mass spectra monitoring NNG in filtrate extracts of *S. noursei* cultures supplemented with natural abundance or ^15^N-Gly (**B** and **C**), ^15^N-Lys (**D** and **E**), or ^13^C-HAA (**F** and **G**).

**
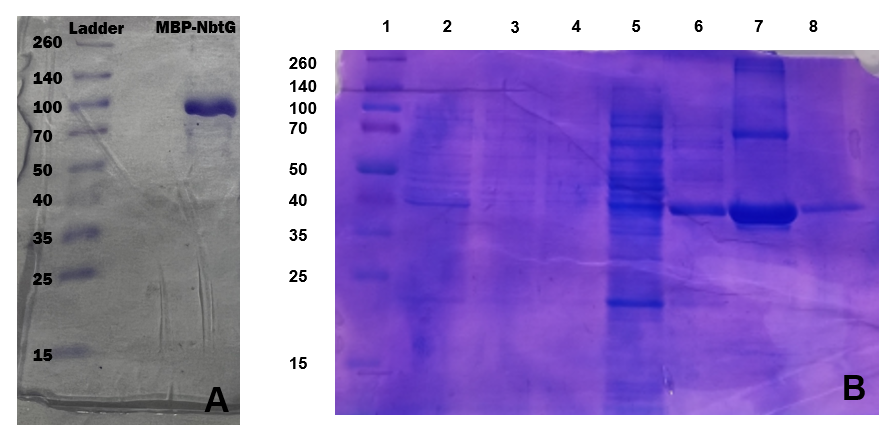
**

**Fig. S2.** SDS-PAGE of Ni^2+^-HTC purified MBP-NbtG (**A**) and Nng39 (**B**). In panel B, the contents of each lane are as follows: Lane 1) molecular weight marker, Lane 2) cell lysate; Lane 3) column flow-through fraction 1; Lane 4) column flow-through fraction 2; Lane 5) column flow-through fraction 3; Lane 6) 20 mM imidazole wash; Lane 7) 250 mM imidazole elution fraction; Lane 8) 1:10 diluted 250 mM imidazole elution fraction.


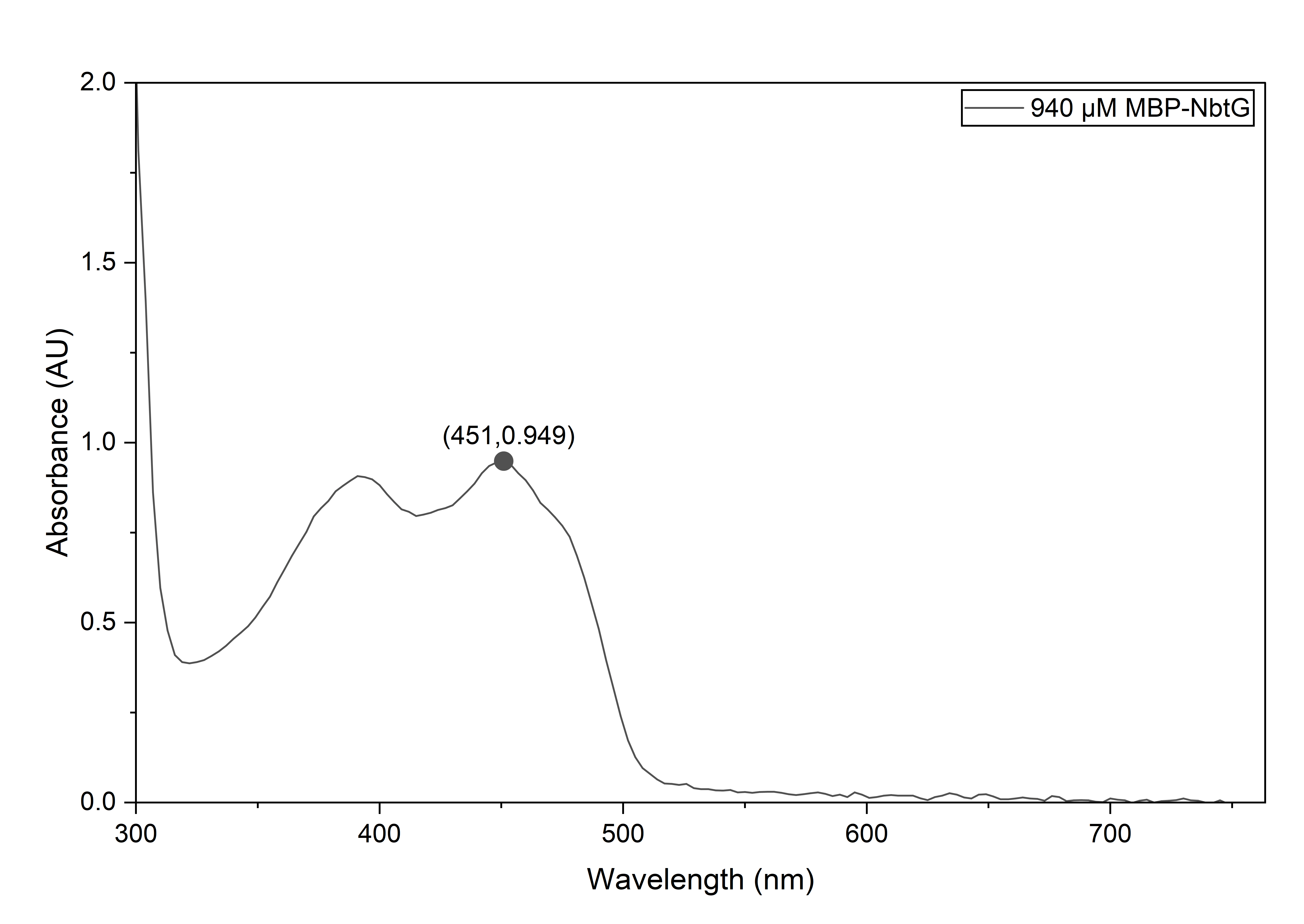


**Fig. S3.** UV-visible absorption spectrum of MBP-NbtG. UV-visible absorption spectrum at 0.1 mm path length of 940 µM purified MBP-NbtG in 50 mM Tris, 150 mM NaCl, 5% glycerol buffer at pH 8.0.


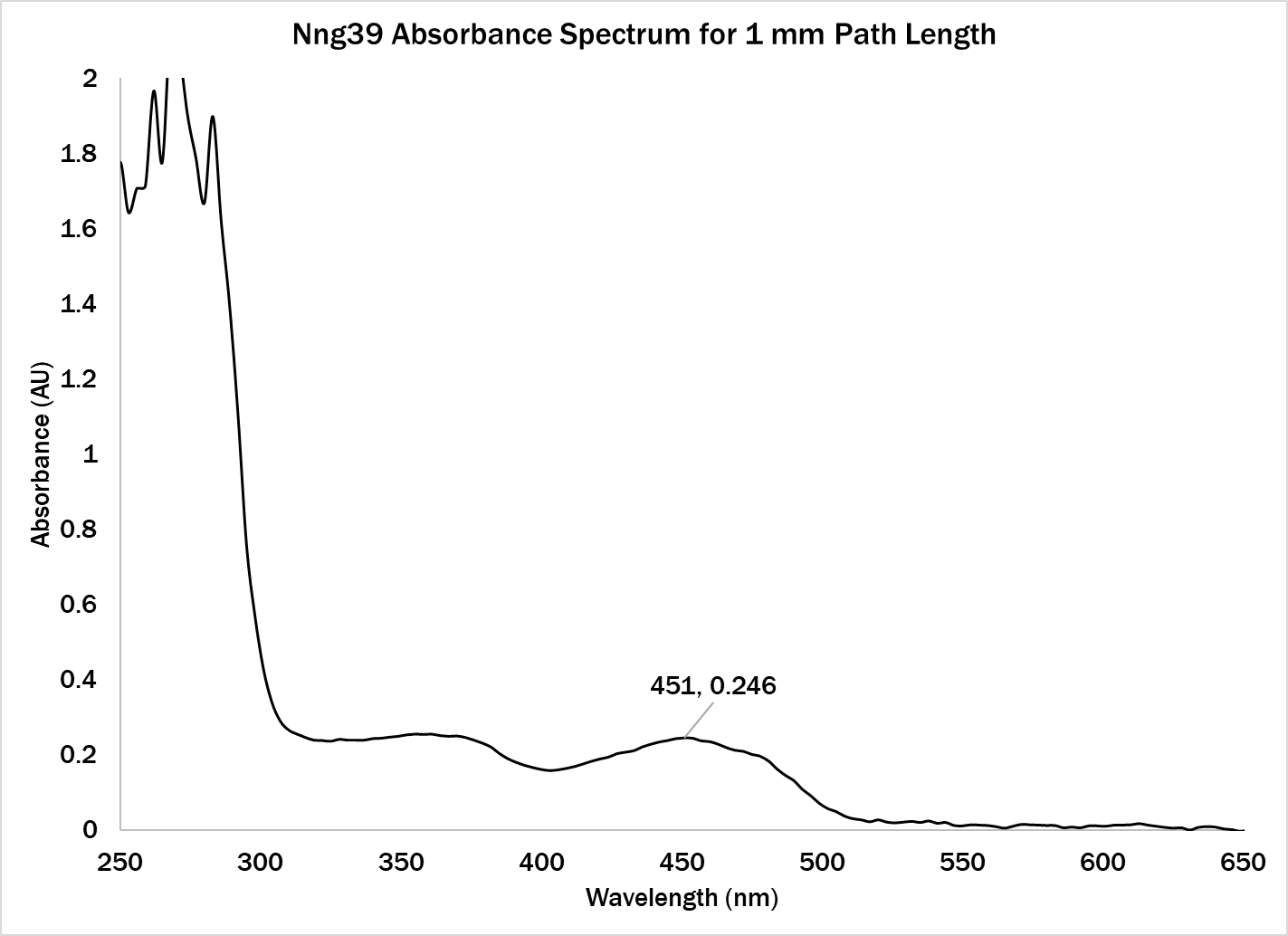


**Fig. S4.** UV-visible absorption spectrum of NngL. UV-visible absorption spectrum at 0.1 mm path length of 340 µM purified Nng39 in 50 mM CHES, 200 mM NaCl, 5% glycerol at pH 9.3.


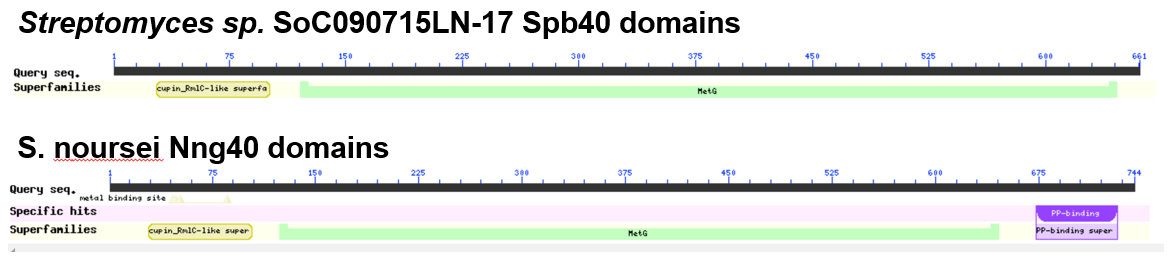


**Fig. S5.** Comparison of predicted domain architecture for Spb40 vs. Nng40 based on the amino acid sequence. An additional domain colored in purple is observed for the long Nng40, which is not observed for Spb40.


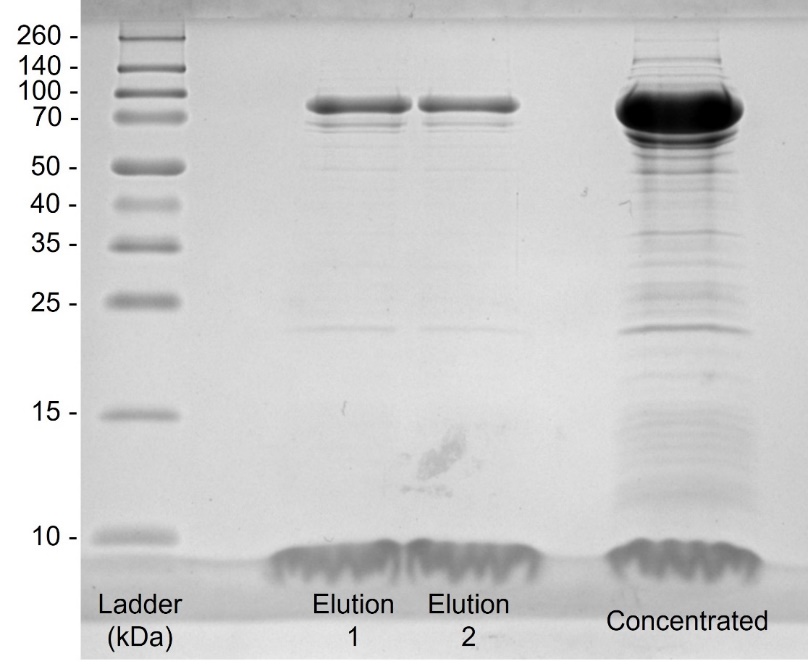
**Fig. S6.** SDS-PAGE of Ni^2+^-HTC purified NngM expressed in *E. coli* BAP1 cells. Lane 1 shows the molecular weight marker while lane 2 show a sample of NngM. The two lanes in **Panel A** are spliced side-by-side from opposite ends of a larger gel, which is shown in **Panel B**.


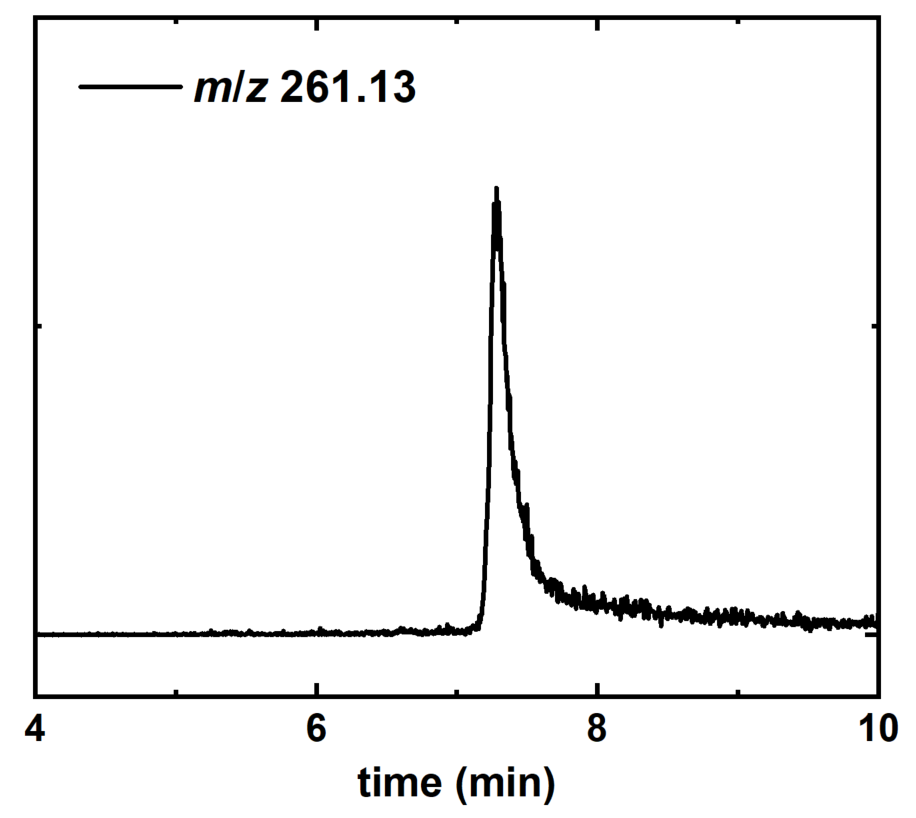


**Fig. S7.** LC-MS extracted ion chromatograms monitoring PPant incorporation in purified Nng40.


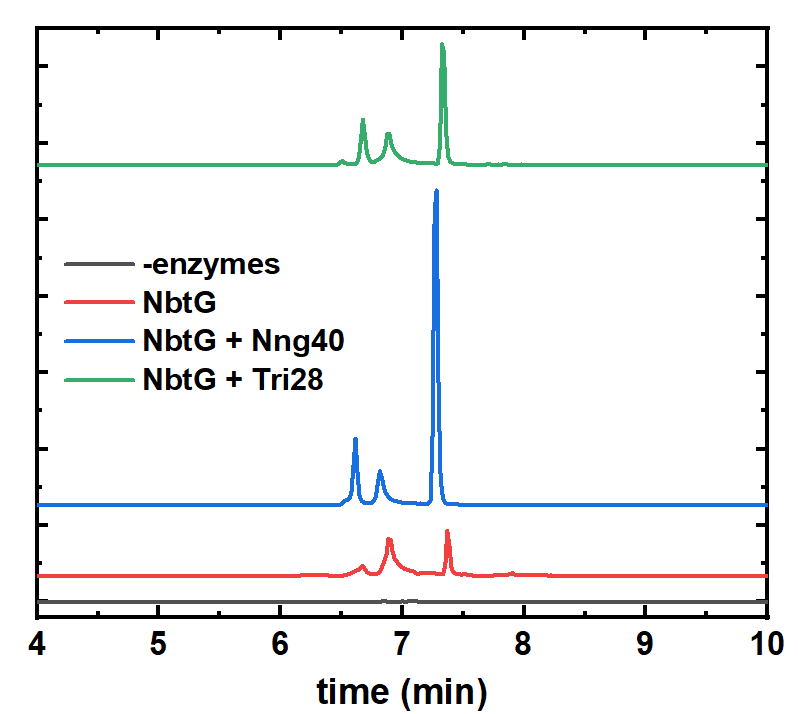


**Fig. S8.** Extracted ion chromatograms monitoring Fmoc-derivatized N^6^-OH-Lys (385.18 +/- 50 ppm) in samples with and without NbtG and Nng40.


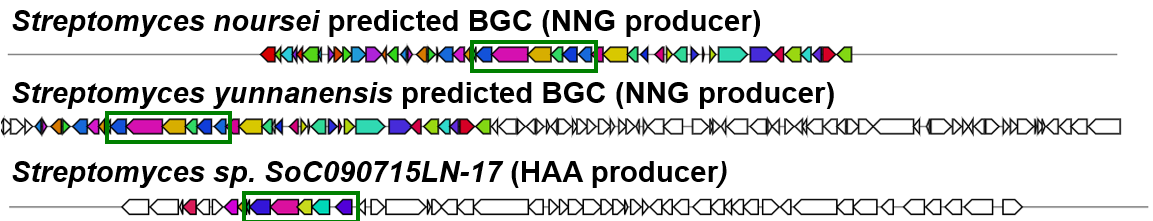


**Fig. S9.** Comparison of antiSMASH-predicted BGCs in the genomes of the NNG-producing bacteria *S. noursei* and *S. yunnanensis* with the characterized BGC from the HAA-producing bacterium Streptomyces sp. SoC090715LN-17. Green boxes show regions containing spb37–41 homologs. Genes with homology to those in the predicted *S. noursei* BGC share the same color, while those with no homology are white.

**
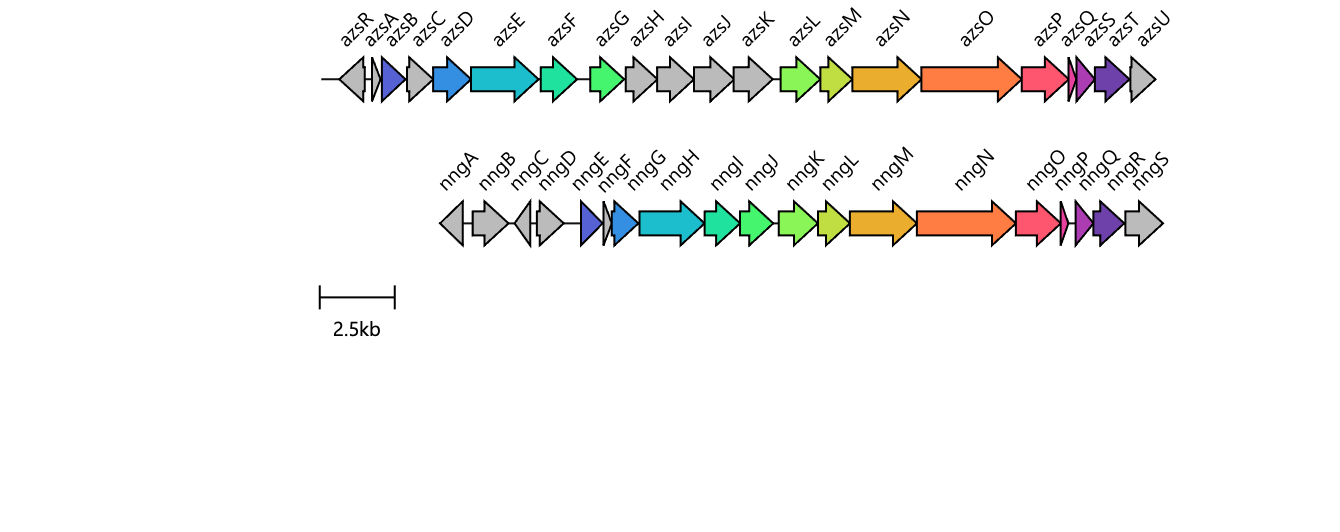
**

**Fig. S10.** Comparison of NNG (nng*) and azaserine (azs*) BGCs (**top**) and the current understanding of the azaserine biosynthetic pathway. Homologous genes within both BGCs are color-matched while genes with no homology between the two clusters are colored grey.

**Fig. S11.** Proposed bifurcation of a common nitrosamine intermediate to form both azaserine and NNG by either non-enzymatic decomposition or oxidation, respectively. Serine residue is not shown for clarity.


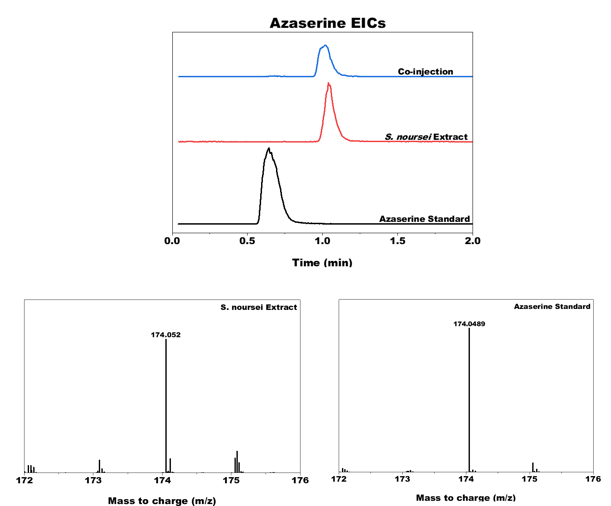


**Fig. S12.** Detection of azaserine in culture extracts of *S. noursei.* **Top panel)** ESI monitoring the [M+H]^+^ of azaserine (174.05 +/- 50ppm) in samples containing azaserine standard (black trace), an *S. noursei* culture extract (red trace) or a co-injection of the two samples (blue trace). **Bottom panels**) MS of standard sample and *S. noursei* culture extract.


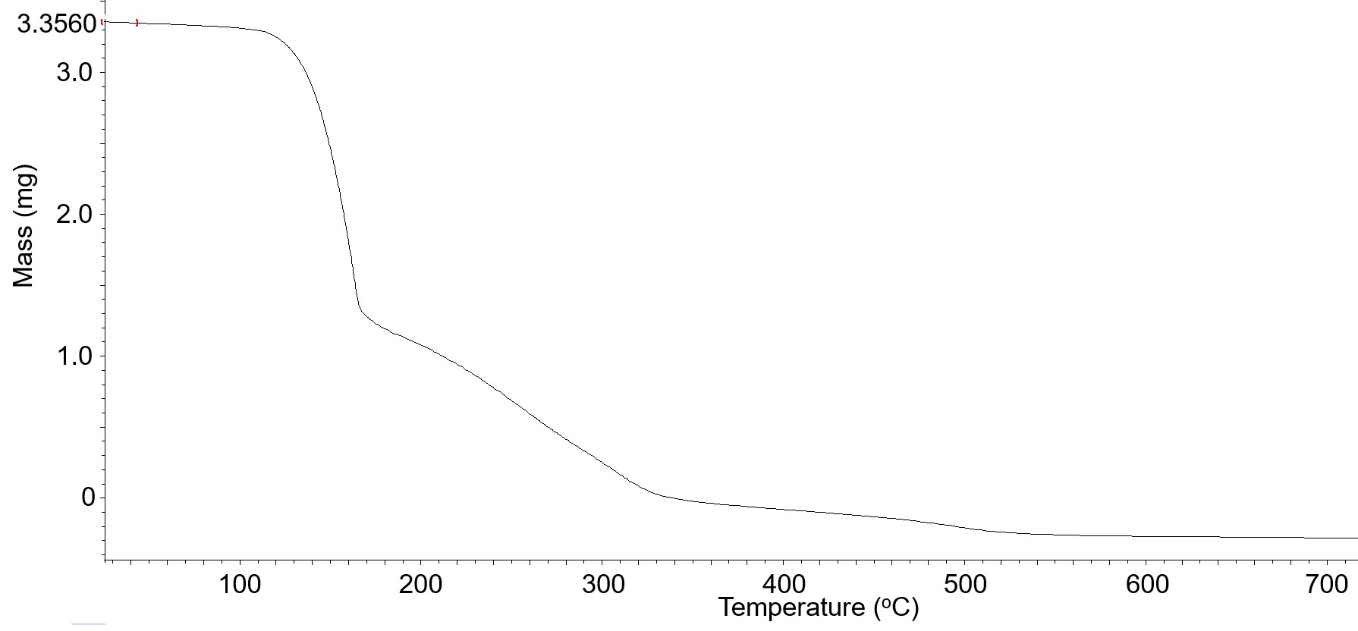


**Fig. S13.** TGA trace of a 3.3560 mg sample of NNG, showing three major mass loss regions. The final position is reproducibly slightly negative, attributed to a heterolytic thermal decomposition mechanism for NNG, which leaves the balance with a residual repulsive charge.


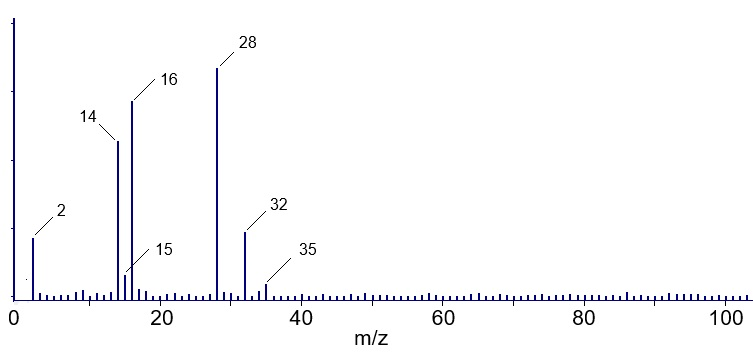


**Fig. S14.** EI mass spectrum of evolved gasses during TGA analysis, showing mass peaks corresponding to H_2_ (2), N (14), O (16), N_2_ (28), and O_2_ (32). The peak at 28 is assigned as N_2_, not CO, due to the absence of atomic carbon (m/z = 12).

| **Table S1.** AntiSMASH predicted BGC in *S. noursei* genome and annotations vs. experimental log_2_-fold differential expression of gene products (stationary vs. log phase of *Streptomyces noursei* JCM-4701). | | | |
| --- | --- | --- | --- |
| **Gene** | **Annotation**  **(*S. noursei* JCM-4071 NCBI accession)** | ***S. noursei* JCM-4701 vs. ATCC 11455 Amino acid sequence coverage (% identity)** | **log_2_ (log_10_)-fold expression of gene product^a^** |
| *orf26* | aromatic-ring-hydroxylating dioxygenase subunit beta |  | ND^b^ |
| *orf25* | hypothetical protein |  | ND |
| *orf24* | aldo/keto reductase |  | ND |
| *orf23* | MmgE/PrpD family protein |  | ND |
| *orf22* | phosphotransferase |  | ND |
| *orf21* | hypothetical protein |  | ND |
| *orf20* | hypothetical protein |  | ND |
| *orf19* | hypothetical protein |  | ND |
| *orf18* | RICIN domain-containing protein |  | ND |
| *orf17* | MFS transporter |  | ND |
| *orf16* | TetR/AcrR family transcriptional regulator  (WP_102922755.1) | 100%  (99.52%) | ND |
| *orf15* | DUF4345 domain-containing protein  (WP_067356884.1) | 100%  (98.54%) | ND |
| *orf14* | LysR family transcriptional regulator  (WP_102922754.1) | 100%  (98.42%) | ND |
| *orf13* | sulfite exporter TauE/SafE family protein  (WP_102924416.1) | 100%  (99.60%) | ND |
| *orf12* | Hypothetical protein  (WP_167390809.1) | 100%  (96.43%) | ND |
| NngS | cytochrome P450  (WP_102922753.1) | 100%  (98.80%) | 6.8 (110) |
| NngR | C45 peptidase  (WP_258017501.1) | 100%  (100%) | 3.9 (15) |
| NngQ | GNAT family N-acetyltransferase  (WP_073447062.1) | 99%  (90.86%) | 8.3 (320) |
| NngP | acyl carrier protein  (WP_102922751.1) | 99%  (91.67%) | 7.1 (140) |
| NngO | AMP-dependent synthetase and ligase  (WP_102922750.1) | 100%  (100%) | 6.2 (74) |
| NngN | Non-ribosomal peptide synthase  (WP_102922749.1) | 100%  (100%) | 7.8 (220) |
| NngM | methionine-tRNA ligase  (WP_073447065.1) | 100%  (98.12%) | 8.9 (480) |
| NngL | FAD dependent oxidoreductase  (WP_102922748.1) | 100%  (98.03%) | 7.7 (210) |
| NngK | l-lysine 6-monooxygenase  (WP_102922747.1) | 100%  (99.31%) | 3.3 (9.8) |
| NngJ | cytochrome P450 oxidoreductase  (WP_102922746.1) | 100%  (98.64%) | 5.8 (56) |
| NngI | acyl-CoA dehydrogenase  (WP_102922745.1) | 100%  (98.73%) | 6.7 (100) |
| NngH | Heme-dioxygenase-like enzyme  (WP_102922744.1) | 100%  (97.65%) | 5.4 (42) |
| NngG | beta-ketoacyl-[acyl-carrier-protein] synthase  (WP_102922743.1) | 100%  (98.31%) | 6.6 (97) |
| NngF | hypothetical protein  (WP_102922742.1) | 100%  (96.74%) | ND |
| NngE | thioesterase II family protein  (WP_258017498.1) | 100%  (99.16%) | 3.8 (14) |
| NngD | patatin-like phospholipase  (WP_102922741.1) | 100%  (97.49%) | 3.7 (13) |
| NngC | hypothetical protein  (WP_102922740.1) | 100%  (98.84%) | ND |
| NngB | NAD(P)/FAD-dependent oxidoreductase  (WP_258017497.1) | 100%  (96.67%) | ND |
| NngA | alpha/beta hydrolase fold containing protein  (WP_073447075.1) | 100%  (97.21%) | 5.5 (45) |
| *orf11* | cold shock protein  (WP_073447076.1) | 100%  (97.65%) | ND |
| *orf10* | helix-turn-helix transcriptional regulator  (WP_180990035.1) | 100%  (100%) | ND |
| *orf9* | class III lanthionine synthetase LanKC  (WP_146051080.1) | 99%  (99.09%) | ND |
| *orf8* | hypothetical protein |  | ND |
| *orf7* | prolyl oligopeptidase  (WP_180990034.1) | 100%  (100%) | ND |
| *orf6* | catechol 1,2-dioxygenase  (WP_102924414.1) | 99%  (99.31%) | ND |
| *orf5* | Xylulose kinase  (WP_102922736.1) | 99%  (97.22%) | ND |
| *orf4* | zinc-containing alcohol dehydrogenase  (WP_102922735.1) | 100%  (99.19%) | 2.6 |
| *orf3* | transcriptional regulator, DeoR family  (WP_258017496.1) | 100%  (99.62%) | ND |
| *orf2* | nitrate/nitrite transporter  (WP_258017495.1) | 100%  (97.05%) | ND |
| *orf1* | erythromycin esterase family protein  (WP_102922734.1) | 100%  (94.25%) | ND |
| ^a^Calculated as counts log_2_(peptide abundance in stationary phase)-log_2_(peptide abundance in exponential phase). Data from ref. 1  ^b^ND = not detected | | | |

| **Table S2. Crystal data and structure refinement for mo_NNG_twin_a.** | |
| --- | --- |
| Identification code | mo_NNG_twin_a |
| Empirical formula | C_2_H_4_N_2_O_4_ |
| Formula weight | 120.07 |
| Temperature/K | 144.10 |
| Crystal system | orthorhombic |
| Space group | Pbcn |
| a/Å | 15.343(7) |
| b/Å | 7.333(4) |
| c/Å | 8.777(4) |
| α/° | 90 |
| β/° | 90 |
| γ/° | 90 |
| Volume/Å^3^ | 987.5(8) |
| Z | 8 |
| ρ_calc_g/cm^3^ | 1.615 |
| μ/mm^‑1^ | 0.158 |
| F(000) | 496.0 |
| Crystal size/mm | 0.128 × 0.037 × 0.019 |
| Radiation | MoKα (λ = 0.71073) |
| 2Θ range for data collection/° | 5.31 to 46.514 |
| Index ranges | -17 ≤ h ≤ 16, -5 ≤ k ≤ 8, -9 ≤ l ≤ 9 |
| Reflections collected | 4169 |
| Independent reflections | 679 [R_int_ = 0.0799, R_sigma_ = 0.0678] |
| Data/restraints/parameters | 679/0/74 |
| Goodness-of-fit on F^2^ | 1.113 |
| Final R indexes [I>=2σ (I)] | R_1_ = 0.0594, wR_2_ = 0.1549 |
| Final R indexes [all data] | R_1_ = 0.0860, wR_2_ = 0.1721 |
| Largest diff. peak/hole / e Å^-3^ | 0.42/-0.26 |

| **Table S3. Fractional Atomic Coordinates (×10^4^) and Equivalent Isotropic Displacement Parameters (Å^2^×10^3^) for mo_NNG_twin_a. U_eq_ is defined as 1/3 of the trace of the orthogonalised U_IJ_ tensor.** | | | | |
| --- | --- | --- | --- | --- |
| **Atom** | ***x*** | ***y*** | ***z*** | **U(eq)** |
| O1 | 1847.5(18) | 2729(4) | 7336(3) | 28.0(9) |
| O2 | 3152.7(17) | 2278(5) | 8259(3) | 30.2(9) |
| N2 | 2865(2) | 1514(6) | 5863(4) | 25.6(10) |
| O3 | 3981.6(19) | 4423(5) | 5385(3) | 30.5(9) |
| O4 | 5108(2) | 2527(5) | 5024(4) | 41.2(11) |
| N1 | 2605(2) | 2220(5) | 7214(4) | 26.2(10) |
| C1 | 3773(3) | 1188(7) | 5590(4) | 25.0(11) |
| C2 | 4291(3) | 2903(7) | 5316(5) | 26.0(11) |

| **Table S4. Anisotropic Displacement Parameters (Å^2^×10^3^) for mo_NNG_twin_a. The Anisotropic displacement factor exponent takes the form: -2π^2^[h^2^a*^2^U_11_+2hka*b*U_12_+…].** | | | | | | |
| --- | --- | --- | --- | --- | --- | --- |
| **Atom** | **U_11_** | **U_22_** | **U_33_** | **U_23_** | **U_13_** | **U_12_** |
| O1 | 24.7(17) | 26(2) | 33.6(17) | 1.0(12) | 3.0(12) | 5.8(14) |
| O2 | 33.9(17) | 34(2) | 22.7(16) | -2.6(12) | -5.9(13) | 1.4(14) |
| N2 | 29(2) | 21(3) | 26.9(18) | -2.5(16) | -2.6(14) | -1.4(17) |
| O3 | 28.8(16) | 13(2) | 49.9(19) | -0.6(14) | 5.8(12) | 2.6(14) |
| O4 | 26.9(19) | 17(3) | 80(3) | -1.9(18) | 8.3(16) | 1.4(15) |
| N1 | 35(2) | 20(3) | 23(2) | 1.7(15) | 1.0(16) | -0.7(18) |
| C1 | 27(2) | 22(3) | 26(2) | -1.3(17) | -1.3(16) | 0.4(19) |
| C2 | 27(2) | 23(3) | 28(2) | -1.8(18) | 1.3(17) | -1(2) |

| **Table S5. Bond Lengths for mo_NNG_twin_a.** | | | | | | |
| --- | --- | --- | --- | --- | --- | --- |
| **Atom** | **Atom** | **Length/Å** |  | **Atom** | **Atom** | **Length/Å** |
| O1 | N1 | 1.225(4) |  | O3 | C2 | 1.213(6) |
| O2 | N1 | 1.245(4) |  | O4 | C2 | 1.309(5) |
| N2 | N1 | 1.354(5) |  | C1 | C2 | 1.507(7) |
| N2 | C1 | 1.435(6) |  |  |  |  |

| **Table S6. Bond Angles for mo_NNG_twin_a.** | | | | | | | | |
| --- | --- | --- | --- | --- | --- | --- | --- | --- |
| **Atom** | **Atom** | **Atom** | **Angle/˚** |  | **Atom** | **Atom** | **Atom** | **Angle/˚** |
| N1 | N2 | C1 | 119.8(3) |  | N2 | C1 | C2 | 113.6(4) |
| O1 | N1 | O2 | 124.5(3) |  | O3 | C2 | O4 | 125.4(4) |
| O1 | N1 | N2 | 118.2(3) |  | O3 | C2 | C1 | 123.5(4) |
| O2 | N1 | N2 | 117.4(3) |  | O4 | C2 | C1 | 111.1(4) |

| **Table S7. Torsion Angles for mo_NNG_twin_a.** | | | | | | | | | | |
| --- | --- | --- | --- | --- | --- | --- | --- | --- | --- | --- |
| **A** | **B** | **C** | **D** | **Angle/˚** |  | **A** | **B** | **C** | **D** | **Angle/˚** |
| N2 | C1 | C2 | O3 | 3.1(6) |  | C1 | N2 | N1 | O1 | 169.6(4) |
| N2 | C1 | C2 | O4 | -177.9(3) |  | C1 | N2 | N1 | O2 | -11.6(6) |
| N1 | N2 | C1 | C2 | -73.7(5) |  |  |  |  |  |  |

| **Table S8. Hydrogen Atom Coordinates (Å×10^4^) and Isotropic Displacement Parameters (Å^2^×10^3^) for mo_NNG_twin_a.** | | | | |
| --- | --- | --- | --- | --- |
| **Atom** | ***x*** | ***y*** | ***z*** | **U(eq)** |
| H2 | 2477.56 | 1256.01 | 5155.56 | 31 |
| H4 | 5391.37 | 3501.95 | 4935.87 | 62 |
| H1A | 3833.38 | 380.61 | 4691.58 | 30 |
| H1B | 4022.37 | 539.11 | 6478.13 | 30 |

**Experimental**

Single crystals of C_2_H_4_N_2_O_4_ **[mo_NNG_twin_a]** were **[]**. A suitable crystal was selected and **[]** on a **Bruker APEX-II CCD** diffractometer. The crystal was kept at 144.10 K during data collection. Using Olex2 [1], the structure was solved with the SHELXT [2] structure solution program using Intrinsic Phasing and refined with the XL [3] refinement package using Least Squares minimisation.

1. Dolomanov, O.V., Bourhis, L.J., Gildea, R.J, Howard, J.A.K. & Puschmann, H. (2009), J. Appl. Cryst. 42, 339-341.
2. Sheldrick, G.M. (2015). Acta Cryst. A71, 3-8.
3. Sheldrick, G.M. (2008). Acta Cryst. A64, 112-122.

**Crystal structure determination of [mo_NNG_twin_a]**

**Crystal Data** for C_2_H_4_N_2_O_4_ (*M*=120.07 g/mol): orthorhombic, space group Pbcn (no. 60), *a* = 15.343(7) Å, *b* = 7.333(4) Å, *c* = 8.777(4) Å, *V*= 987.5(8) Å^3^, *Z* = 8, *T* = 144.10 K, μ(MoKα) = 0.158 mm^-1^, *Dcalc* = 1.615 g/cm^3^, 4169 reflections measured (5.31° ≤ 2Θ ≤ 46.514°), 679 unique (*R*_int_ = 0.0799, R_sigma_ = 0.0678) which were used in all calculations. The final *R*_1_ was 0.0594 (I > 2σ(I)) and *wR*_2_ was 0.1721 (all data).

**Refinement model description**

Number of restraints - 0, number of constraints - unknown.

Details:

1. Fixed Uiso
 At 1.2 times of:
 All C(H,H) groups, All N(H) groups
 At 1.5 times of:
 All O(H) groups
2.a Secondary CH2 refined with riding coordinates:
 C1(H1A,H1B)
2.b Aromatic/amide H refined with riding coordinates:
 N2(H2)
2.c Idealised tetrahedral OH refined as rotating group:
 O4(H4)


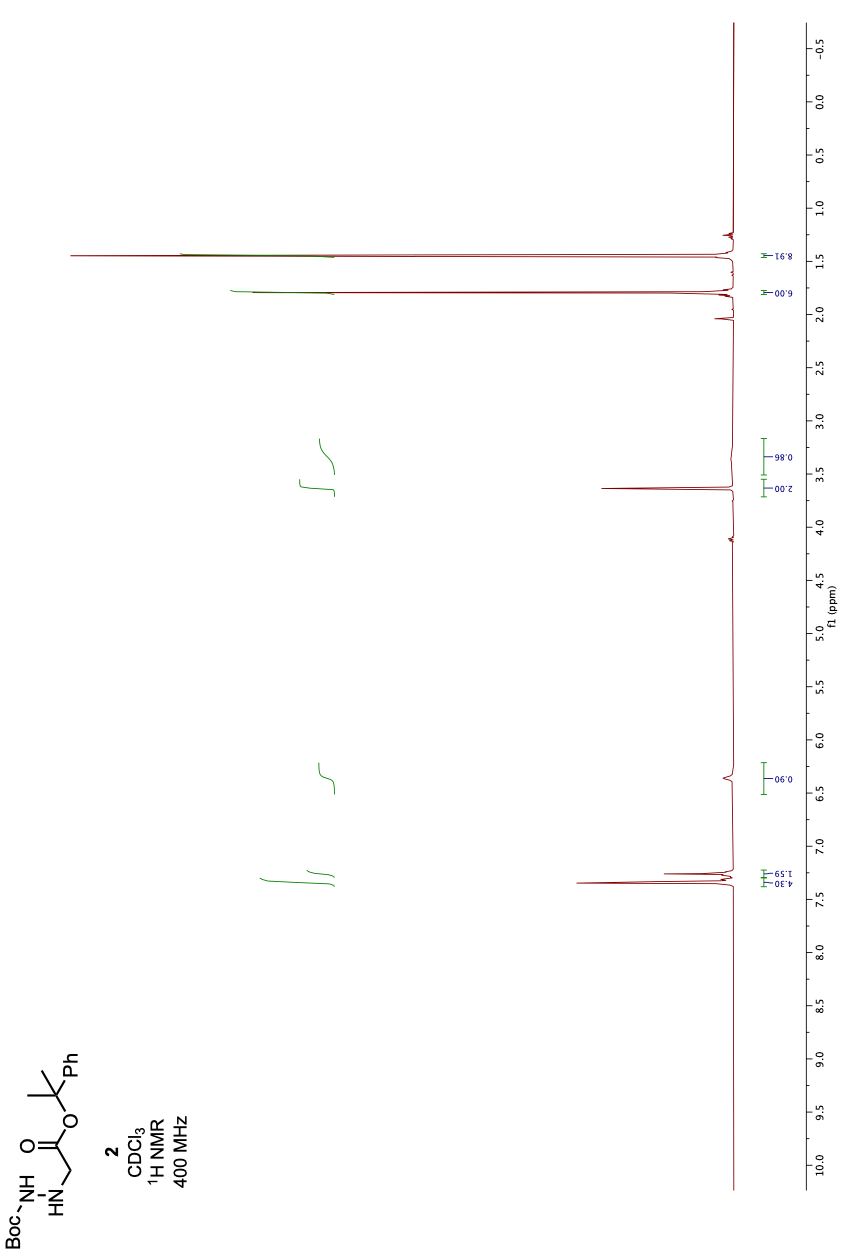


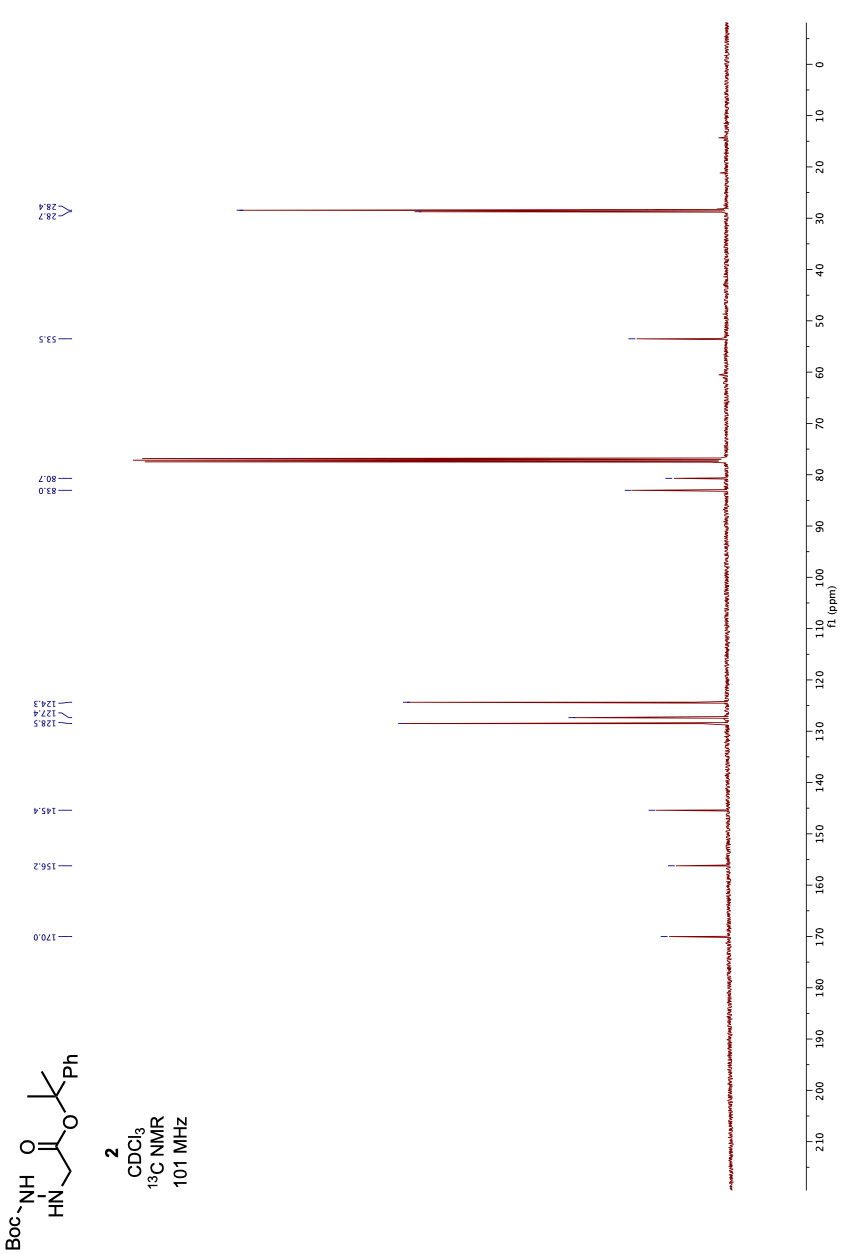


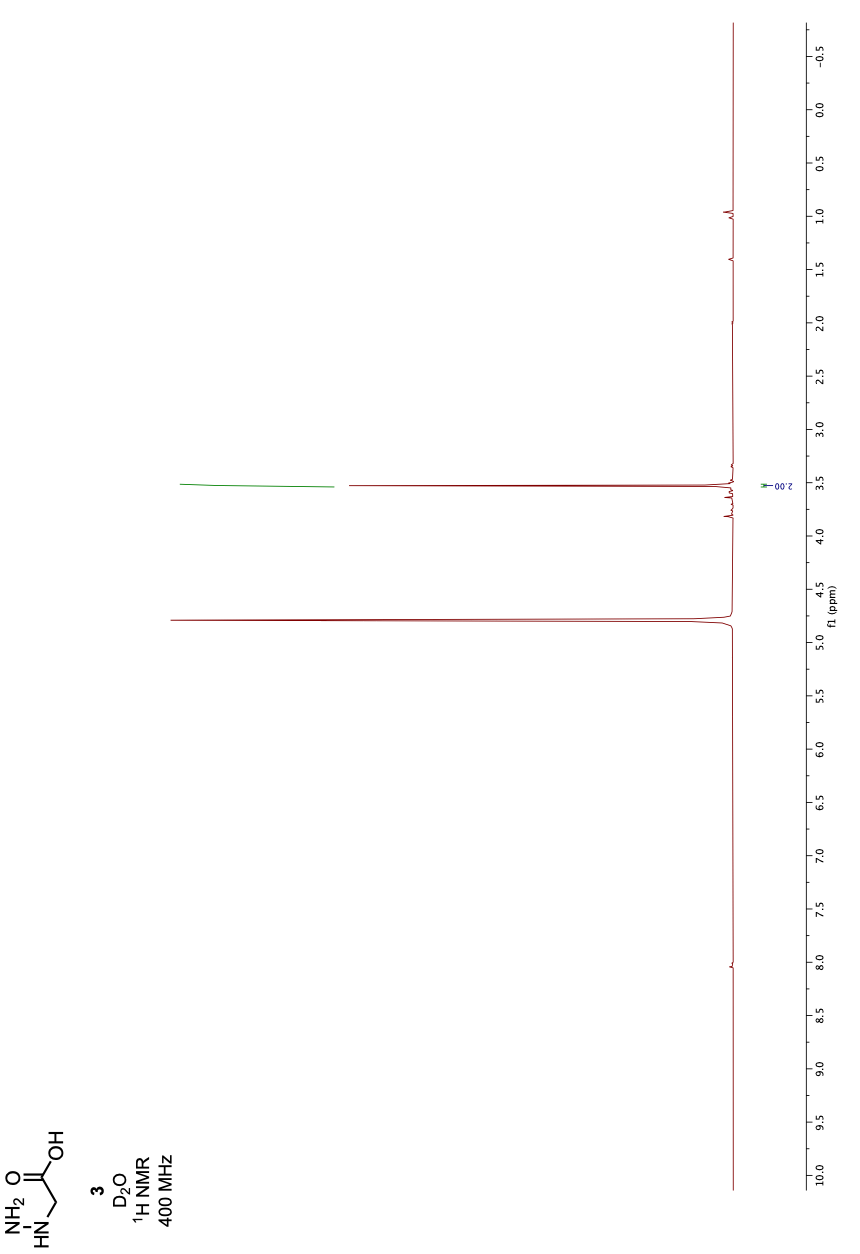


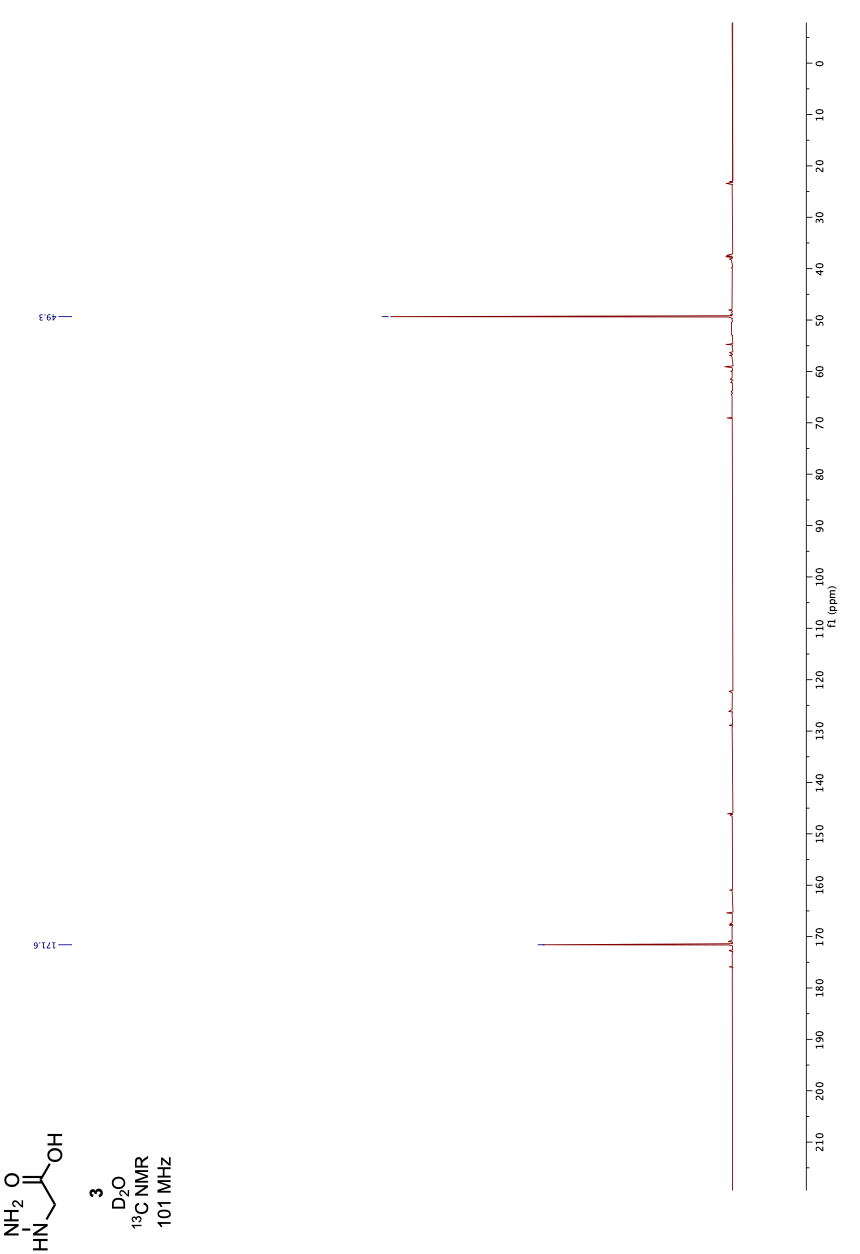


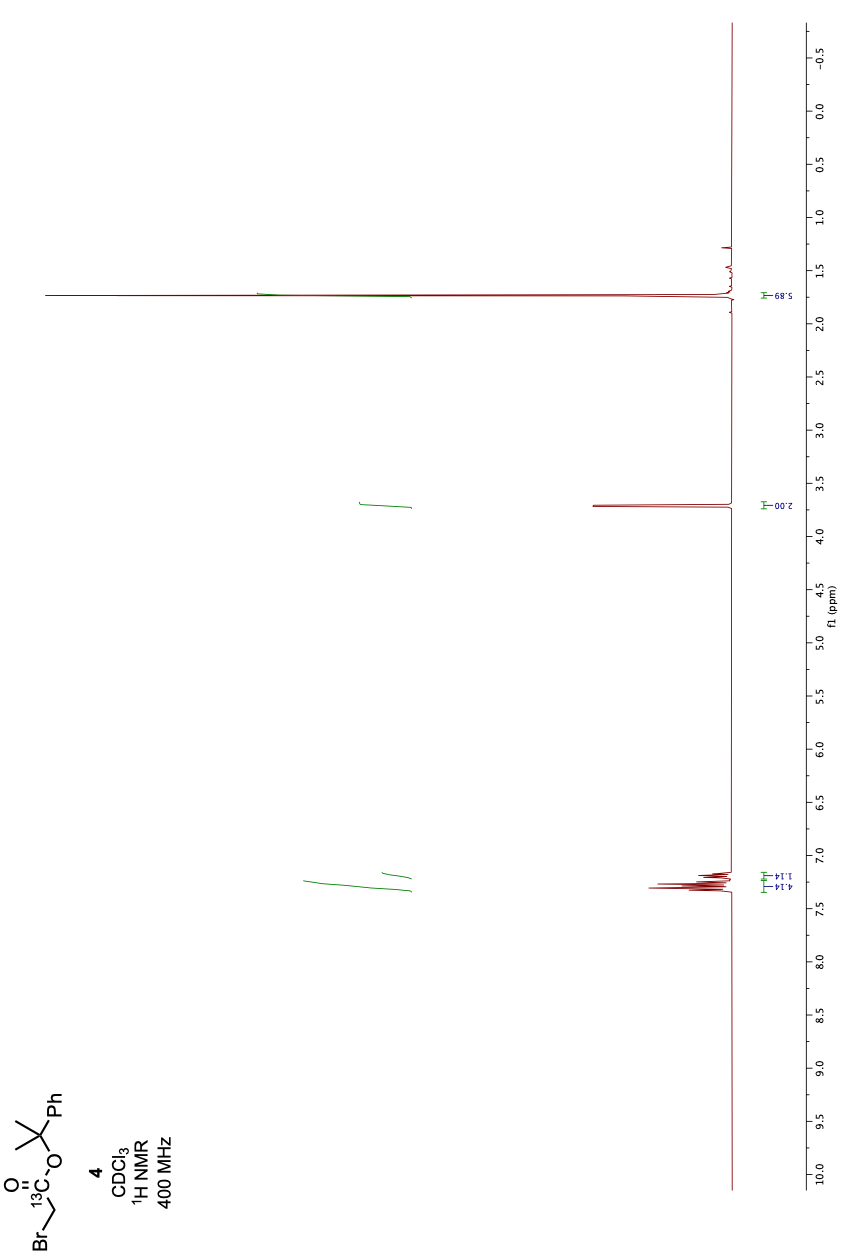


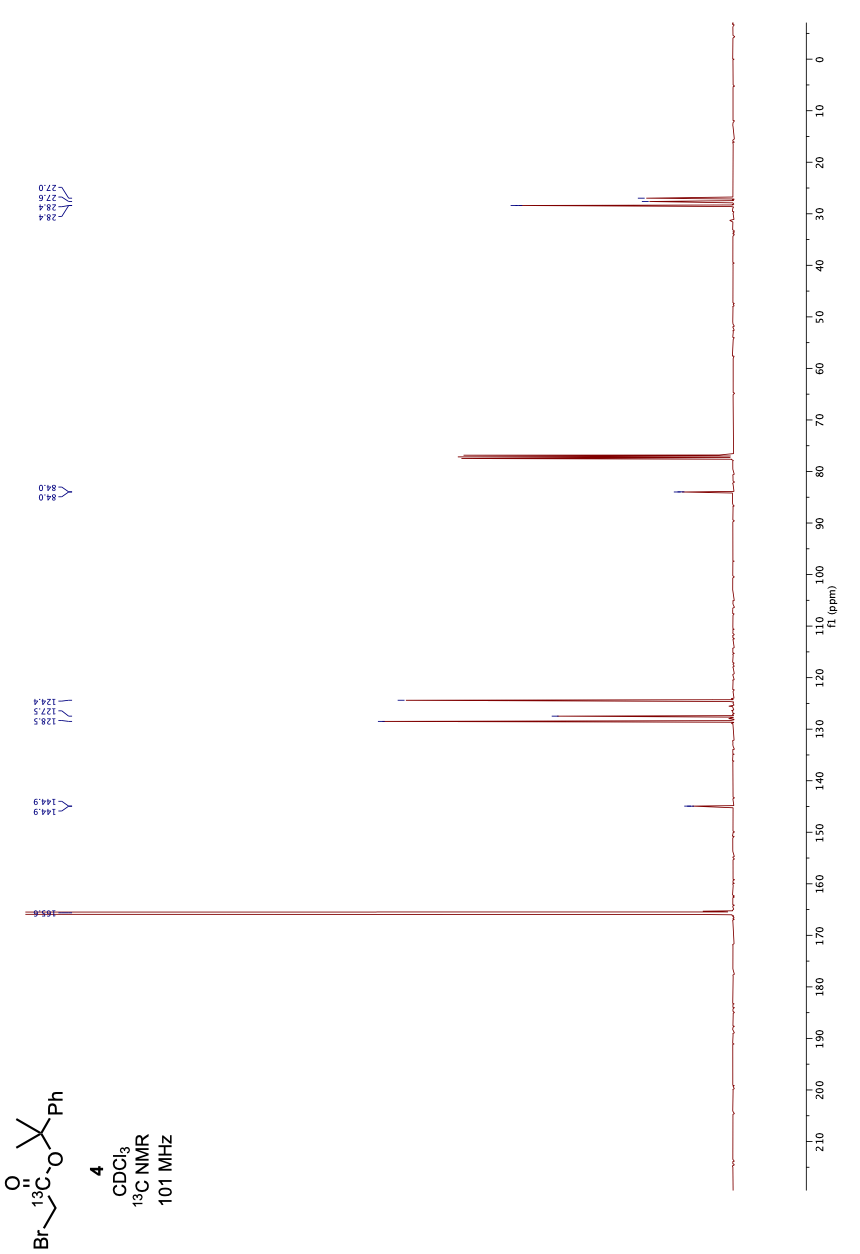


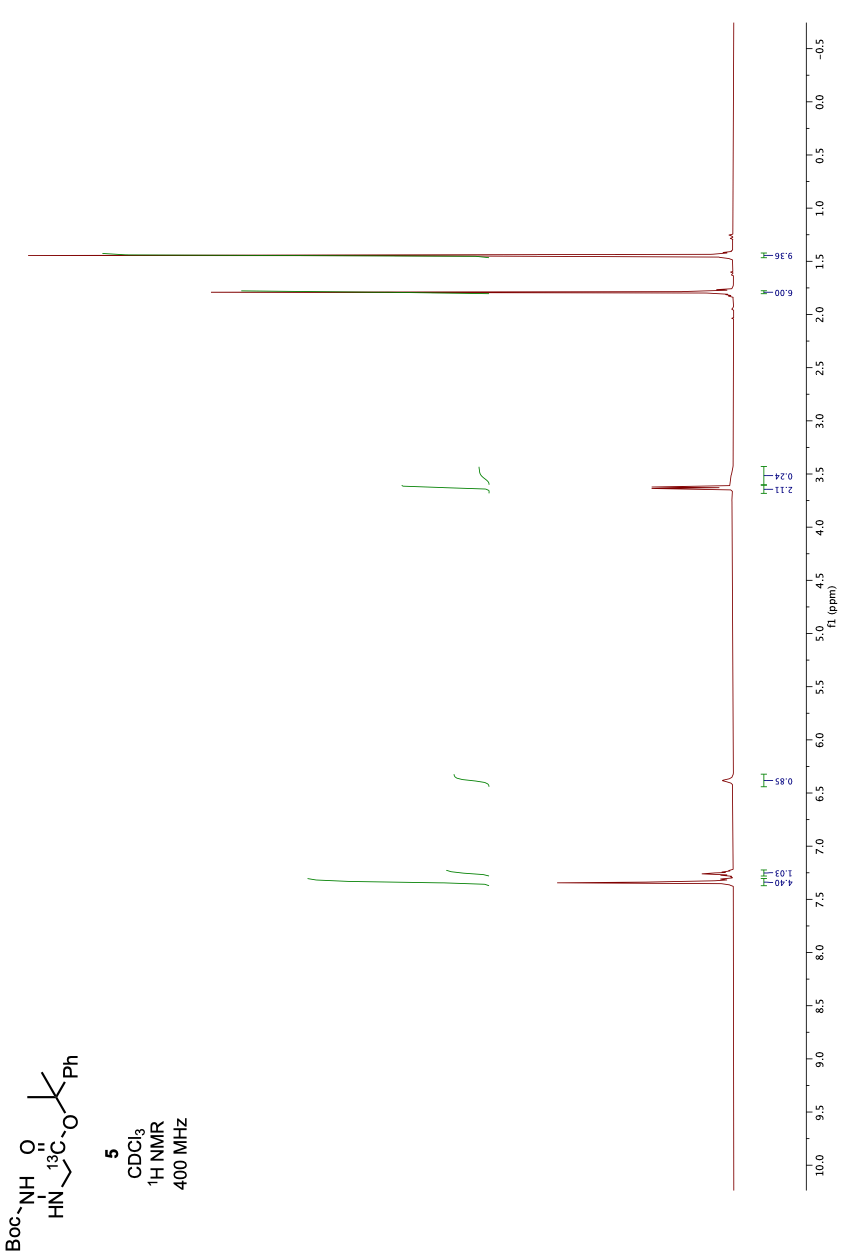


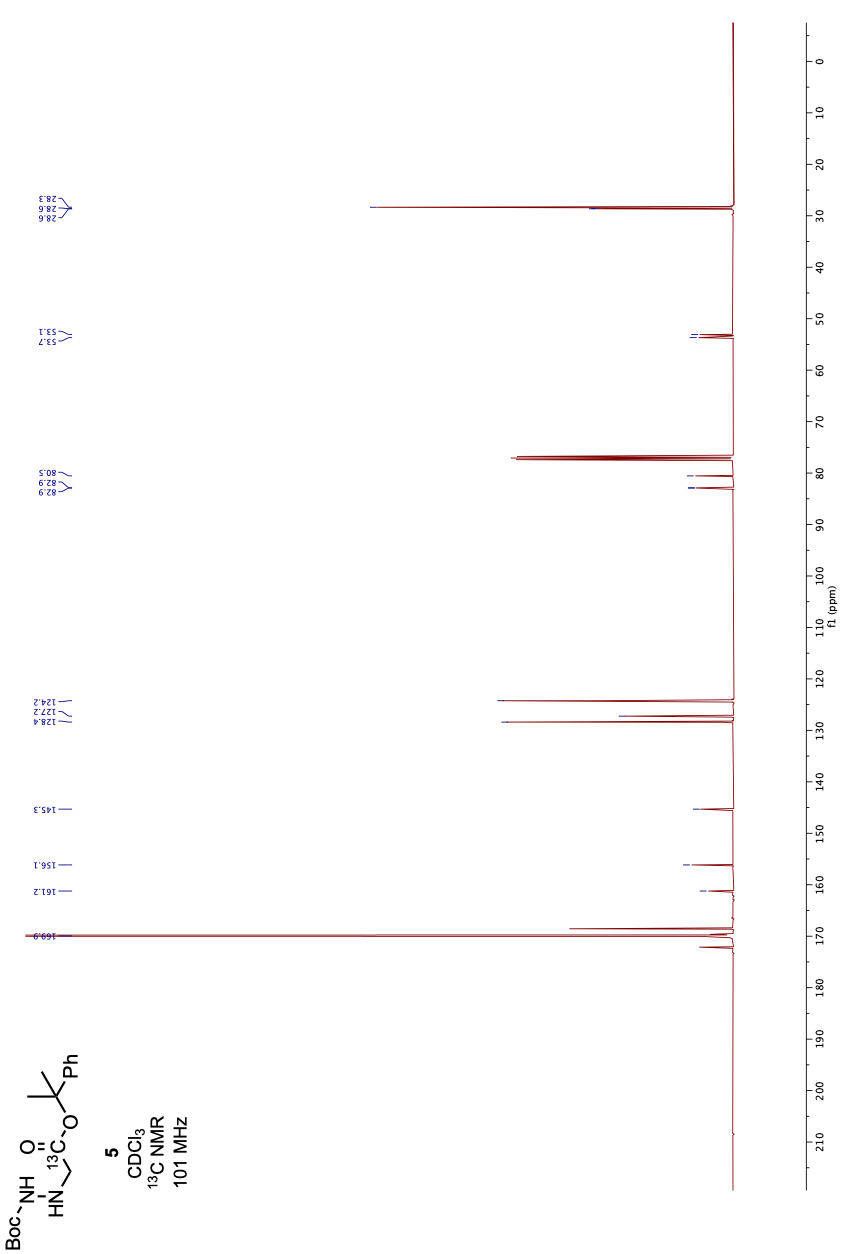


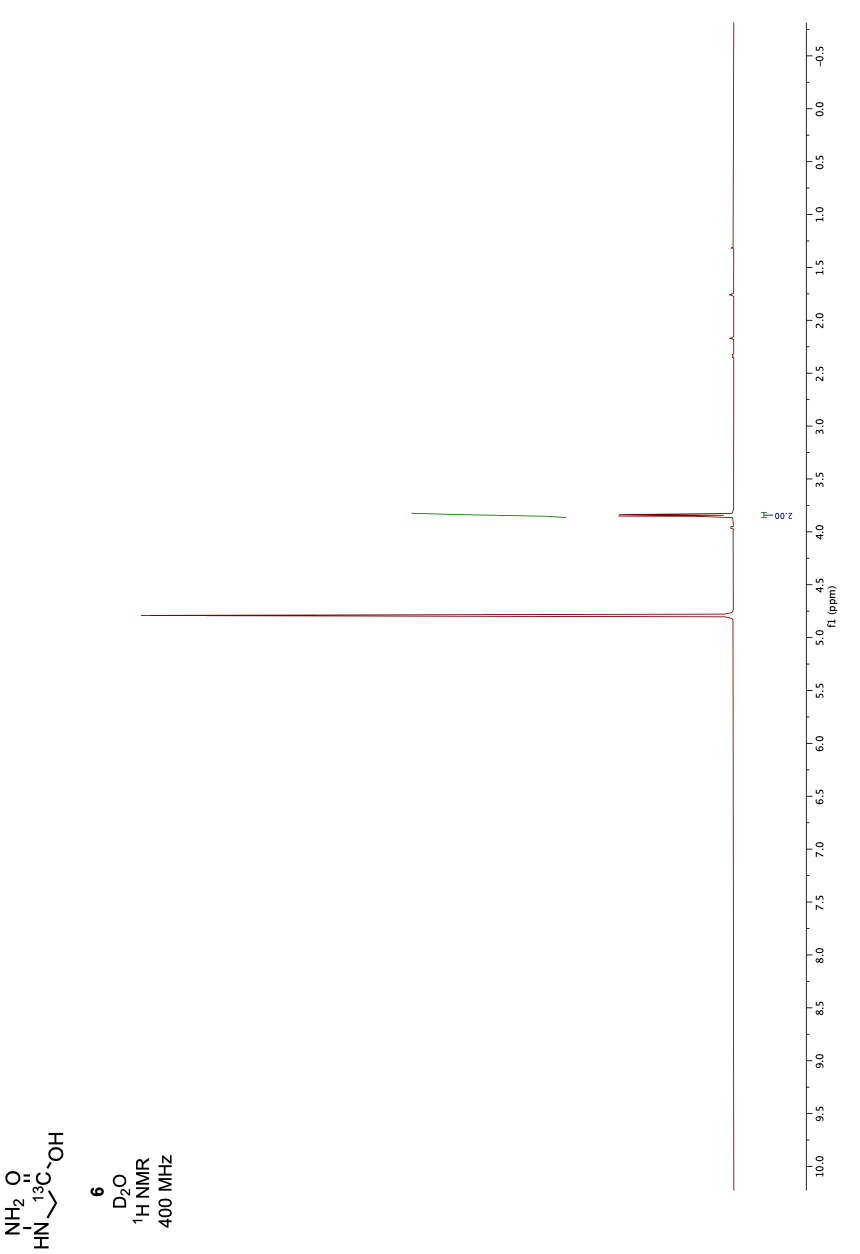


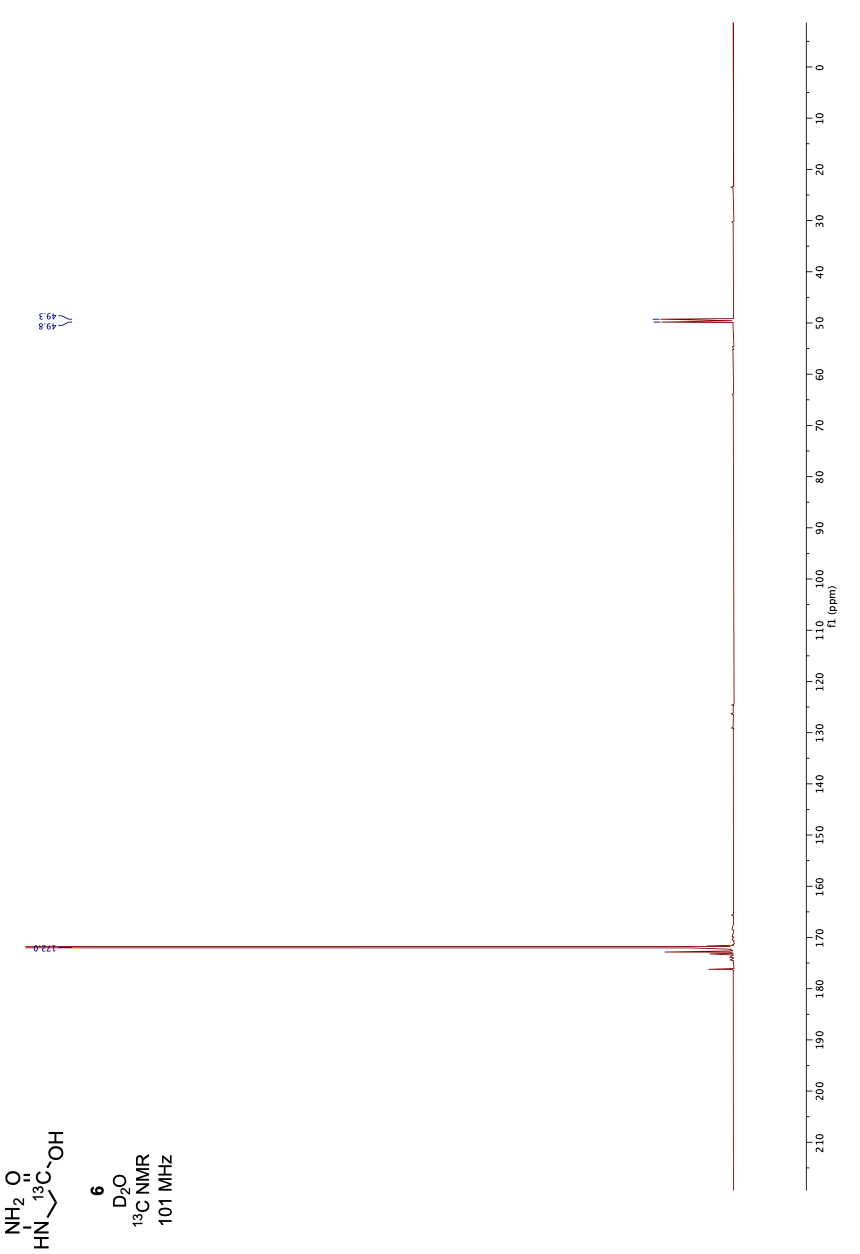


**References**

(1) Respondek, T.; Cueny, E.; Kodanko, J. J. Cumyl Ester as the C-Terminal Protecting Group in the Enantioselective Alkylation of Glycine Benzophenone Imine. *Org. Lett.* **2012**, *14* (1), 150–153. <https://doi.org/10.1021/ol202939g>.

(1) Graham, D. E.; Spain, J. C.; Parry, R. J.; Hettich, R. L.; Mahan, K. M.; Klingeman, D. M.; Giannone, R. J.; Gulvick, C. A.; Fida, T. T. *Nitration enzyme toolkit for the biosynthesis of energetic materials (ORNL/SPR-2017/498)*; Oak Ridge National Lab, Oak Ridge, TN, 2018.
