# Supplementary material for: Hydrazinoacetic acid is a biosynthetic precursor of the bacterially produced nitramine, N-nitroglycine
Source: Appl Environ Microbiol. 2026 May 20;92(6):e00631-26. doi: 10.1128/aem.00631-26 (PMC13274452; doi:10.1128/aem.00631-26)

checkCIF/PLATON report

Structure factors have been supplied for datablock(s) mo_nng_twin_a

THIS REPORT IS FOR GUIDANCE ONLY. IF USED AS PART OF A REVIEW PROCEDURE FOR PUBLICATION, IT SHOULD NOT REPLACE THE EXPERTISE OF AN EXPERIENCED CRYSTALLOGRAPHIC REFEREE.

No syntax errors found. CIF dictionary Interpreting this report

Datablock: mo_nng_twin_a

Bond precision: C-C = 0.0070 A Wavelength=0.71073

Cell:	a=15.343(7)	b=7.333(4)	c=8.777(4)
	alpha=90	beta=90	gamma=90
Temperature:	144 K		

	Calculated	Reported
Volume	987.5(8)	987.5(8)
Space group	P b c n	P b c n
Hall group	-P 2n 2ab	-P 2n 2ab
Moiety formula	C2 H4 N2 O4	C2 H4 N2 O4
Sum formula	C2 H4 N2 O4	C2 H4 N2 O4
Mr	120.07	120.07
Dx,g cm-3	1.615	1.615
Z	8	8
Mu (mm-1)	0.158	0.158
F000	496.0	496.0
F000'	496.37	
h,k,lmax	17,8,9	17,8,9
Nref	707	679
Tmin,Tmax	0.993,0.997	0.495,0.745
Tmin'	0.980	

Correction method= # Reported T Limits: Tmin=0.495 Tmax=0.745
AbsCorr = MULTI-SCAN

Data completeness= 0.960   Theta(max)= 23.257

R(reflections)= 0.0594(488)   wR2(reflections)= 0.1721(679)

S = 1.113   Npar= 74

The following ALERTS were generated. Each ALERT has the format
test-name_ALERT_alert-type_alert-level.
Click on the hyperlinks for more details of the test.

Alert level B

```
THETM01_ALERT_3_B  The value of sine(theta_max)/wavelength is less than 0.575
            Calculated sin(theta_max)/wavelength =    0.5556
```

Author Response: These were very small needle crystals and diffracted poorly. Yet 0.9A resolution is sufficient for compound identification and obtainment of accurate bond metrics.

Alert level C

```
PLAT029_ALERT_3_C _diffrn_measured_fraction_theta_full value Low .      0.960 Why?
PLAT088_ALERT_3_C Poor Data / Parameter Ratio ...................       9.18 Note
PLAT340_ALERT_3_C Low Bond Precision on  C-C Bonds ..............      0.007 Ang.
PLAT767_ALERT_4_C INS Embedded LIST 6 Instruction Should be LIST 4     Please Check
PLAT906_ALERT_3_C Large K Value in the Analysis of Variance ......      2.520 Check
PLAT911_ALERT_3_C Missing FCF Refl Between Thmin & STh/L=    0.556         28 Report
                0  2  0,   0  4  0,   0  6  0,   0  8  0,   1  1  0,   1  3  0,
                1  5  0,   1  7  0,   2  2  0,   2  4  0,   2  6  0,   3  3  0,
                3  5  0,   3  7  0,   4  4  0,   4  6  0,   5  5  0,   6  4  0,
                6  6  0,   7  5  0,   0  2  1,   0  6  1,   0  8  1,   1  7  1,
                0  4  2,   0  6  2,   1  6  2,   0  6  3,
```

Alert level G

```
PLAT007_ALERT_5_G Number of Unrefined Donor-H Atoms .............          2 Report
              H2      H4
PLAT909_ALERT_3_G Percentage of I>2sig(I) Data at Theta(Max) Still        52% Note
PLAT913_ALERT_3_G Missing # of Very Strong Reflections in FCF ....          1 Note
                0  2  0,
PLAT953_ALERT_1_G Reported (CIF) and Actual (FCF) Hmax Differ by .          1 Units
PLAT969_ALERT_5_G The 'Henn et al.' R-Factor-gap value ..........      2.367 Note
              Predicted wR2: Based on SigI**2  7.27 or SHELX Weight 15.47
PLAT978_ALERT_2_G Number C-C Bonds with Positive Residual Density.          0 Info
PLAT994_ALERT_1_G SHELXL .ins Contains no or MERG 0 Instruction ..          ! Note
```

---

0 **ALERT level A** = Most likely a serious problem - resolve or explain
1 **ALERT level B** = A potentially serious problem, consider carefully
6 **ALERT level C** = Check. Ensure it is not caused by an omission or oversight
7 **ALERT level G** = General information/check it is not something unexpected

```
 2 ALERT type 1 CIF construction/syntax error, inconsistent or missing data
 1 ALERT type 2 Indicator that the structure model may be wrong or deficient
 8 ALERT type 3 Indicator that the structure quality may be low
 1 ALERT type 4 Improvement, methodology, query or suggestion
 2 ALERT type 5 Informative message, check
```

It is advisable to attempt to resolve as many as possible of the alerts in all categories. Often the minor alerts point to easily fixed oversights, errors and omissions in your CIF or refinement strategy, so attention to these fine details can be worthwhile. It is up to the individual to critically assess their own results and, if necessary, seek expert advice.

PLATON version of 15/01/2026; check.def file version of 02/01/2026

---

duplicate check

No duplication found

Datablock mo_nng_twin_a - ellipsoid plot

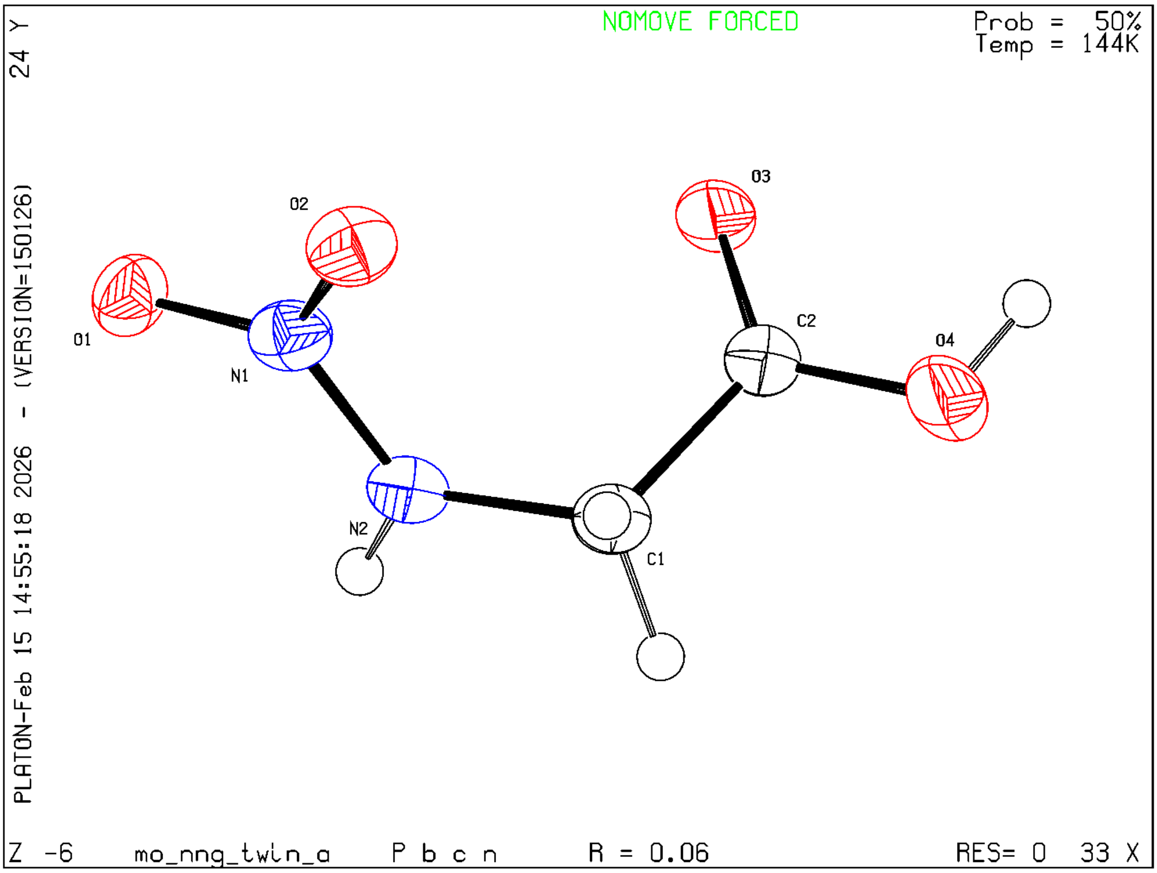

Supplement: File S2 — Crystallographic tables and TGA-MS data. [file aem.00631-26-s0002.pdf]
